# Purinergic receptors P2Y12R and P2X7R: potential targets for PET imaging of microglia phenotypes in multiple sclerosis

**DOI:** 10.1186/s12974-017-1034-z

**Published:** 2017-12-22

**Authors:** Wissam Beaino, Bieneke Janssen, Gijs Kooij, Susanne M. A. van der Pol, B. van Het Hof, Jack van Horssen, Albert D. Windhorst, Helga E. de Vries

**Affiliations:** 1 Department of Radiology and Nuclear Medicine, VU University Medical Center, Amsterdam, The Netherlands; 2 Department of Molecular Cell Biology and Immunology, VUmc MS Center Amsterdam, Amsterdam Neuroscience, VU University Medical Center, Amsterdam, The Netherlands

**Keywords:** Multiple sclerosis, P2Y12R, P2X7R, PET imaging, Neuroinflammation, Microglia

## Abstract

**Background:**

Microglia are major players in the pathogenesis of multiple sclerosis (MS) and may play a dual role in disease progression. The activation status of microglia in vivo is highly dynamic and occurs as a continuum, with the pro-inflammatory and anti-inflammatory phenotypes on either end of this spectrum. Little is known about in vivo dynamics of microglia phenotypes in MS due to the lack of diagnostic tools. Positron emission tomography (PET) imaging is a powerful non-invasive technique that allows real-time imaging of microglia activation phenotypes in the central nervous system, depending on the availability of selective PET tracers. Our objective is to investigate and characterize the expression of the purinergic receptors P2Y12R and P2X7R as potential targets for PET tracer development and subsequent PET imaging in order to evaluate the dynamics of microglia status in vivo.

**Methods:**

We used immunohistochemical analysis to explore the expression of P2Y12R and P2X7R in experimental autoimmune encephalomyelitis (EAE) post-mortem tissues and different stages of well-characterized MS lesions. We evaluated by quantitative real-time polymerase chain reaction the expression of P2Y12R and P2X7R in human polarized microglia, and we performed autoradiography binding assay with radiolabeled P2Y12R and P2X7R antagonists using MS and rat EAE tissues.

**Results:**

Here, we demonstrate that P2X7R is associated with a pro-inflammatory phenotype of human microglia in vitro, and is highly expressed in microglia in MS lesions as well as during the peak of EAE. In contrast, P2Y12R was associated with an anti-inflammatory phenotype in human microglia in vitro and was expressed at lower levels in active inflammatory MS lesions compared to normal-appearing white matter (NAWM) and similarly in EAE, while its expression increased in the remission phase of EAE. Binding of radiolabeled tracers specific for P2Y12R and P2X7R on ex vivo tissues validated the value of these receptors as PET imaging targets for microglia phenotypes in vivo.

**Conclusion:**

Our results suggest that P2Y12R and P2X7R are excellent targets for PET imaging to discriminate distinct microglia phenotypes in MS. Ultimately, this may provide insight into the role of microglia in disease progression and monitor novel treatment strategies to alter microglia phenotype.

**Electronic supplementary material:**

The online version of this article (10.1186/s12974-017-1034-z) contains supplementary material, which is available to authorized users.

## Background

Multiple sclerosis (MS) is an inflammatory autoimmune disease of the central nervous system (CNS) characterized by massive immune cell infiltration, glia activation, severe demyelination, and neurodegeneration [1]. In the disease progression, microglia play a profound role through the secretion of pro-inflammatory mediators, myelin phagocytosis, and antigen presentation. However, recent insights indicate that in MS, microglia also exert protective and anti-inflammatory effects by the production of neuroprotective factors, thereby playing a role in disease resolution [2–4]. Microglia are highly dynamic cells that are constantly sensing their microenvironment [5, 6]. The activation status of microglia in vivo is highly dynamic and occurs as a continuum, with pro-inflammatory cytotoxic microglia on one end of the spectrum and anti-inflammatory, repair promoting microglia on the other side [7, 8]. In the experimental autoimmune encephalomyelitis (EAE) model, the well-established animal model for MS, it has been shown that pro-inflammatory microglia and macrophages contribute to disease development by secreting inflammatory cytokines and recruitment of cytotoxic T cells into the CNS [9, 10]. On the other hand, anti-inflammatory microglia and macrophages are predominant in the recovery phase where they contribute to phagocytosis of myelin debris and associated axonal regeneration [11, 12]. This protective role was corroborated by several studies showing that injection of macrophages polarized towards a more anti-inflammatory phenotype inhibited or ameliorated EAE associated clinical signs in mice [13, 14].

The role of microglia in MS pathology and its contribution to disease progression remains unclear. The development of new treatments for MS relies heavily on improved understanding of neuroinflammation and the role of activated microglia in the disease evolution. Many questions are still pending concerning the role of pro- and anti-inflammatory microglia in MS and how immunomodulatory therapy affects the state of microglia activation. Getting insights into the molecular dynamics and polarization of activated microglia in vivo is only possible through a highly sensitive and non-invasive imaging technique as positron emission tomography (PET). Current developments in PET imaging of activated microglia are centered around targeting translocator protein (TSPO), a mitochondrial protein overexpressed in activated microglia [15]. TSPO imaging provided valuable information in detecting the presence of active neuroinflammation, but failed to discriminate between pro- and anti-inflammatory states of microglia and was subjected to significant level of polymorphism between different subjects which limited its utility [16]. Hence, there is an urgent need for new PET imaging tools and new target receptors for imaging activated microglia in neuroinflammation.

Ionotropic and metabotropic receptors P2X7R and P2Y12R, respectively, are expressed on microglia and are involved in microglia response to brain tissue damage and neurodegeneration [9]. P2X7R is a purinergic, ATP binding, receptor expressed on immune cells such as monocytes, macrophages, and microglia [17]. In addition, P2X7R plays an important role in activating the inflammasome and subsequent release of IL-1β [18–20]. P2Y12R belong to the family of G protein-coupled receptor, and studies have shown that P2Y12R is only expressed on microglia and not peripheral or infiltrated macrophages in the brain [21, 22].

In this study, we investigated P2X7R and P2Y12R receptors as potential targets for PET imaging of pro- and anti-inflammatory microglia in well-characterized MS tissue samples and the EAE model.

Our data indicate that P2X7R and P2Y12R are promising selective targets for PET imaging of microglia phenotypes in vivo. Visualization of microglia activation and the dynamics of their activation status will provide valuable insights into the status and role of microglia during MS disease progression. Furthermore, it may contribute to a better understanding of the relation between microglia activation status and neurodegeneration, and in the development and evaluation of new immunomodulatory and disease-modifying therapies.

## Methods

### Reagents

Chemicals were obtained from commercial sources and used without further purification. Solvents were purchased from Sigma-Aldrich (Zwijndrecht, The Netherlands), MERCK (Darmstadt, Germany) and Biosolve (Valkenswaard, The Netherlands) and used as received unless stated otherwise. Tetrahydrofuran (THF) was first distilled from LiAlH_4_ and then stored on 3 Å molecular sieves. [^3^H]methyl nosylate stock solution (717 MBq/mL in toluene, molar radioactivity of 3.08 GBq.μmol^−1^) was obtained from Novandi Chemistry AB (Södertälje, Sweden) and dried at 60 °C under an argon flow prior to use.

### Radioligand synthesis

#### [^3^H]A-740003

[^3^H]A-740003 was synthesized in a similar manner as previously described for [^11^C]A-740003 [23]. Briefly, desmethyl precursor was reacted with [^3^H]methyl nosylate in the presence of tetrabutylammonium hydroxide for 16 h at room temperature. HPLC purification was performed on a Reprospher 100 C18-DE (50 × 8 mm, 5 μm) column (Dr. Maisch GmbH, Ammerbuch, Germany) using MeCN/H_2_O/diisopropylamine (22:78:0.1, *v/v/v*) as eluent at a flow rate of 3 mL.min^−1^. Fractions containing product (*t*_R_ = 16 min) were collected and formulated in 1.5 mL 96% EtOH. Radioactivity concentration (10.9 kBq.μL^−1^) was determined by liquid scintillation counting (LSC) (LKB/Wallac 1219 Rackbeta, Mount Waverley, Australia) using 5 mL of Optiphase “Highsafe 3” scintillation liquid (PerkinElmer, Waltham, MA, USA). The identity of the product was confirmed with analytical HPLC by coinjection of the product and non-labeled A-740003 (*t*_R_ = 10 min), and the (radiochemical) purity was > 99%. Analytical isocratic HPLC was performed on an Xbridge C18 5 μm (100 × 4.6 mm) column (Waters, Milford, MA, USA) using MeCN/H_2_O (30:70, *v*/*v*) as eluent at a flow rate of 1 mL.min^−1^.

#### [^11^C]P2Y12R antagonist

Ethyl 6-(3-(3-((5-chlorothiophen-2-yl)sulphonyl)[^11^C]ureido)azetidin-1-yl)-5-cyano-2-methylnicotinate, as well as precursors ethyl 6-(3-aminoazetidin-1-yl)-5-cyano-2-methylnicotinate and 5-chlorothiophene-2-sulphonyl azide were synthesized as previously described [24]. Hereafter, we will refer to this antagonist as [^11^C]P2Y12R-ant. Briefly, [^11^C]CO was transferred to a vial containing a solution of the precursors, chloro(1,5-cyclooctadiene)rhodium(I) dimer and triphenylphosphine in dry THF. The reaction mixture was heated at 100 °C for 5 min, and subsequently, the excess of the azide precursor was quenched, simultaneously evaporating most of THF. After semi-preparative HPLC purification on an Altima C18 5 μm (250 × 10 mm) column (Grace, Columbia, MD, USA) using MeCN/H_2_O/TFA (55:45:0.1, *v/v/v*) as eluent at a flow rate of 4 mL.min-1, ethyl 6-(3-(3-((5-chlorothiophen-2-yl)sulphonyl)[^11^C]ureido)azetidin-1-yl)-5-cyano-2-methylnicotinate (*t*_R_ = 16 min) was formulated in 1 mL of EtOH and diluted with 5 mL of 7.09 mM NaH_2_PO_4_ in saline. The tracer was obtained in a decay-corrected radiochemical yield (RCY) of 6 ± 2% (*n* = 2; calculated from [^11^C]CO_2_ at end of bombardment) with a radiochemical purity > 99%, a molar radioactivity of 37 ± 4 GBq.μmol^−1^ (*n* = 2). The identity of the product was confirmed with analytical HPLC on a Platinum C18 5 μm (250 × 4.6 mm) column (Grace, Columbia, MD, USA) using MeCN/H_2_O/trifluoroacetic acid (TFA) (45:55:0.1, *v/v/v*) as eluent at a flow rate of 1 mL.min^−1^ by coinjection of the product and non-labeled reference compound (*t*_R_ = 12 min).

### Human brain tissue

Brain tissue from three controls and eight MS patients were used in this study. Patient details are presented in Additional file 1: Table S1. Brain tissue samples were obtained from the Netherlands Brain Bank (coordinator Dr. Huitinga, Amsterdam, The Netherlands). The Netherlands Brain Bank received permission to perform autopsies for the use of tissue and for access to medical records for research purposes from the Ethical Committee of the VU University Medical Center, Amsterdam, The Netherlands. All patients and controls, or their next of kin, had given informed consent for autopsy and use of brain tissue for research purposes.

### EAE model

We used EAE tissues acquired from an independent study performed in our laboratory and the acute experimental autoimmune encephalomyelitis (EAE) was induced as follows. Eight- to 11-week-old male Lewis rats (200 g) obtained from Harlan (Zeist, The Netherlands), as described before [25]. Rats were injected s.c. with 20 μg synthetic myelin basic protein 63–88 peptide, 500 μg Mycobacterium tuberculosis type 37HRa (Difco, Detroit, MI, USA), and 50 μl complete Freund’s adjuvant (CFA) (Difco) supplemented with PBS to reach a volume of 100 μl. Rats were examined daily (weight and clinical disease) and graded on a scale from 1 to 5 for neurological signs. Clinical disease in EAE animals was apparent around day 10 post-immunization with a maximum clinical score between days 14 and 15. Animals were housed under standard laboratory conditions with water and food ad libitum. CFA-immunized animals were used as a control. Animals were sacrificed at day 14 dpi (peak of the disease) and day 20 dpi (recovery phase, end of clinical signs).

### Immunohistochemistry

For human tissue staining, air-dried frozen sections (5 μm) were fixed with acetone (10 min at RT). For single immunohistochemistry staining of proteolipid protein (PLP), MHC-II, purinergic receptor P2X7R, and P2Y12R, we used the protocol described previously by Vogel et al. [4] using Envision-HRP (Dako) and 3,3′-diaminobenzidine (Dako) as detection method.

For immunofluorescence staining on human tissue, sections were fixed with acetone then blocked for non-specific binding with goat serum (10%) for 20 min at RT. Sections were then incubated with the primary antibody (anti-P2Y12R or anti-P2X7R) in PBS/1% serum overnight at 4 °C. Sections were then washed with PBS three times for 5 min and incubated with fluorescence-labeled secondary antibody (1/400) in PBS/1% serum or for 1 h at RT. Sections were then washed three times for 5 min with PBS and blocked with mouse serum (10%) for 20 min at RT followed by incubation with anti-MHC-II or anti-CD31 or anti-GFAP antibody in PBS/1% BSA for 1 h at RT. After three times of washes for 5 min, a fluorescently labeled secondary antibody was added in PBS/1% BSA for 1 h at room temperature. Nuclei were stained with Hoechst (1/1000) for 1 min in the dark. After a final wash for three times for 5 min with PBS, sections were mounted with coverslips using aqueous mounting media Mowiol.

For rat tissue immunofluorescence double staining, air-dried frozen sections (7 μm) were used. Tissues from three different EAE animals at day 14 post-immunization and three different EAE animals at day 20 post-immunization, and three CFA control rat brains were used. Sections were fixed with acetone for 10 min at RT and blocked for non-specific binding using BSA 2%. Sections were then incubated with a mix of both primary antibodies overnight at 4 °C or 1 h at room temperature. Sections were then washed three times in PBS/0.05% Tween-20 and then incubated with fluorescently labeled secondary antibodies and DAPI (1/1000) for nuclear staining for 1 h at RT. Sections were then washed three times for 5 min with PBS/0.05% Tween-20. Sections were mounted with coverslips using aqueous mounting media Mowiol.

Fluorescence images were acquired using Leica DM6000 microscope equipped with a motorized stage. Whole-slide low-zoom images were acquired using ×10 objective and merged by tile stitching to get the final image. For fluorescence quantification and comparison, sections were stained in a single run and images were collected in a single session with the same exposure time between different areas and different slides. Bright field images were acquired on a Zeiss bright field microscope equipped with a colored camera. Images were analyzed using Leica LAS AF or Zen 2012 software. The list of antibodies used for immunohistochemistry and immunofluorescence are described in Additional file 1: Table S3.

### Human microglia isolation

Human microglia isolation and preparation was performed as described elsewhere [3]. In brief, 5 to 10 g of brain white matter was obtained at autopsy, and microglia isolation procedure was performed within 4 to 24 h. Single-cell suspension was prepared using 0.05% trypsin (Sigma, St Louis, MO). Cells were then filtered through a 100-μm nylon mesh (BD Bioscience, Durham, NC) and centrifuged, and the pellet is resuspended in a gradient buffer (3.56 g/L Na_2_HPO_4_·2H_2_O, 0.78 g/L NaH_2_PO_4_·H_2_O, 8 g/L NaCl, 4 g/L KCl, 2 g/L d-(+)-glucose, and 2 g/L BSA, pH 7.4) and centrifuged for 35 min at 1200×*g*. The cell debri-myelin layer was removed. The cell pellet was treated with red blood cell lysis buffer (8.3 g/L NH_4_CL and 1 g/L KHCO_3_, pH 7.4) for 10 min at 4 °C. Cells were then suspended and cultured in DMEM/Ham nutrient mixture F10 (1:1), 1% penicillin-streptomycin-glutamine, and 25 ng/mL granulocyte-macrophage colony-stimulating factor (GM-CSF is only added for the first 2 days). After 7 days, microglia were polarized into a pro-inflammatory status by priming with recombinant IFN-γ (1 × 103 U/mL) (U-Cytech, Utrecht, The Netherlands) for 24 h, followed by addition of *Escherichia coli* LPS (10 ng/mL) (LPS-EB ultrapure; Invitrogen, San Diego, CA) to the medium for 24 h. To induce anti-inflammatory microglia, cells were stimulated with IL-4 (10 ng/mL) (Immunotools, Friesoythe, Germany) for 48 h. Untreated cells were referred to as M0.

### RNA isolation and real-time quantitative polymerase chain reaction

All oligonucleotides were synthesized by Ocimum Biosolutions (Ocimum Biosolutions, Ijsselstein, The Netherlands). RNA was isolated using triazol. cDNA was synthesized with the Reverse Transcription System kit (Promega, Madison, WI, USA) following manufacturer’s guidelines as described previously [26]. QPCR reactions were performed on an Applied Biosystems ViiA 7 machine with the SYBR Green method (Applied Biosystems, Carlsbad, CA, USA). Obtained expression levels of transcripts were normalized to GAPDH and PolRF2 expression levels. Here, we report only the results normalized to GAPDH as we did not observe any difference of normalized data between GAPDH and PolRF2. All primer sequences are listed in Additional file 1: Table S2.

### Western blot

Cells were lysed with RIPA lysis buffer containing protease inhibitors for 1 h on ice. The cells were then centrifuged at 14,500 rpm for 15 min, the pellet was discarded, and the supernatant was collected. Electrophoresis was performed under denaturating conditions on 10% SDS polyacrylamide gel. The proteins were transferred to a nitrocellulose membrane. Blots were saturated in Odyssey buffer/PBS (1:1) for 1 h at RT. The blots were incubated with primary antibodies Rabbit anti-human P2X7R (Alomone, Jerusalem BioPark (JBP), Israel), or Rabbit anti-human P2Y12R (Ananspec, LIEGE Science Park, Belgium) in Odyssey buffer/PBS (1:1) + Tween-20 (0.1%) overnight at 4 °C followed by fluorescently labeled secondary antibody (Odyssey) in Odyssey buffer/PBS (1:1) + Tween-20 (0.1%) for 1 h at room temperature. Blots were imaged using Odyssey imager. Blot were then washed and stained with goat anti-human β-actin (Santa Cruz, Heidelberg, Germany) antibody following the same procedure as above. Western blot relative quantification was realized by calculating the ratio of the integrated intensity of the band of the protein of interest over the integrated intensity of the band of β-actin.

### Autoradiography

For autoradiography tissues from three non-neurological controls, three active, two chronic active, and three chronic inactive MS lesions, and from three different EAE animals at day 14 post-immunization and three different EAE animals at day 20 post-immunization, and three CFA control rat brains were used. Tissue cryosections (20 μm) of acute EAE and human MS were mounted on glass slides, air dried and stored at −80 °C until use. Sections were washed in assay buffer (50 mM Tris-HCl, pH 7.4) for three times 5 min and then dried under an air flow. Sections were incubated with [^11^C]P2Y12R-ant (10 nM) for 30 min at room temperature, or incubated with [^3^H]A740003 for 1 h and 30 min at room temperature. Sections were then washed three times for 90 s with Tris-HCl (50 mM, pH 7.4) and dipped twice in deionized water. Sections were dried under an air flow and exposed to phosphor screen BAS-IP TR 2025 (for tritum-labeled tracer) or phosphor screen BAS-IP SR 2025 (for carbon-11-labeled tracer) (General Electric, Eindhoven The Netherlands) for 1 h for [^11^C]P2Y12R or 48 h for [^3^H]A740003. Phosphor screen were then imaged using Typhoon FLA 7000 imager (General Electric, Eindhoven, The Netherlands). The intensity of the signal was quantified using image Quant software (General Electric, Eindhoven, The Netherlands). Three independent experiments were performed.

### Statistical analysis

All data are presented as mean ± SD. Groups were compared using the two-tailed Student *t* test (normal), and Mann-Whitney (non-normal) and analysis were done with Graphpad prism 5 (San Diego, CA, USA). *p* values of less than 0.05 were considered statistically significant.

## Results

### Dynamic of expression of P2Y12R and P2X7R in human polarized microglia

**Fig. 1:**
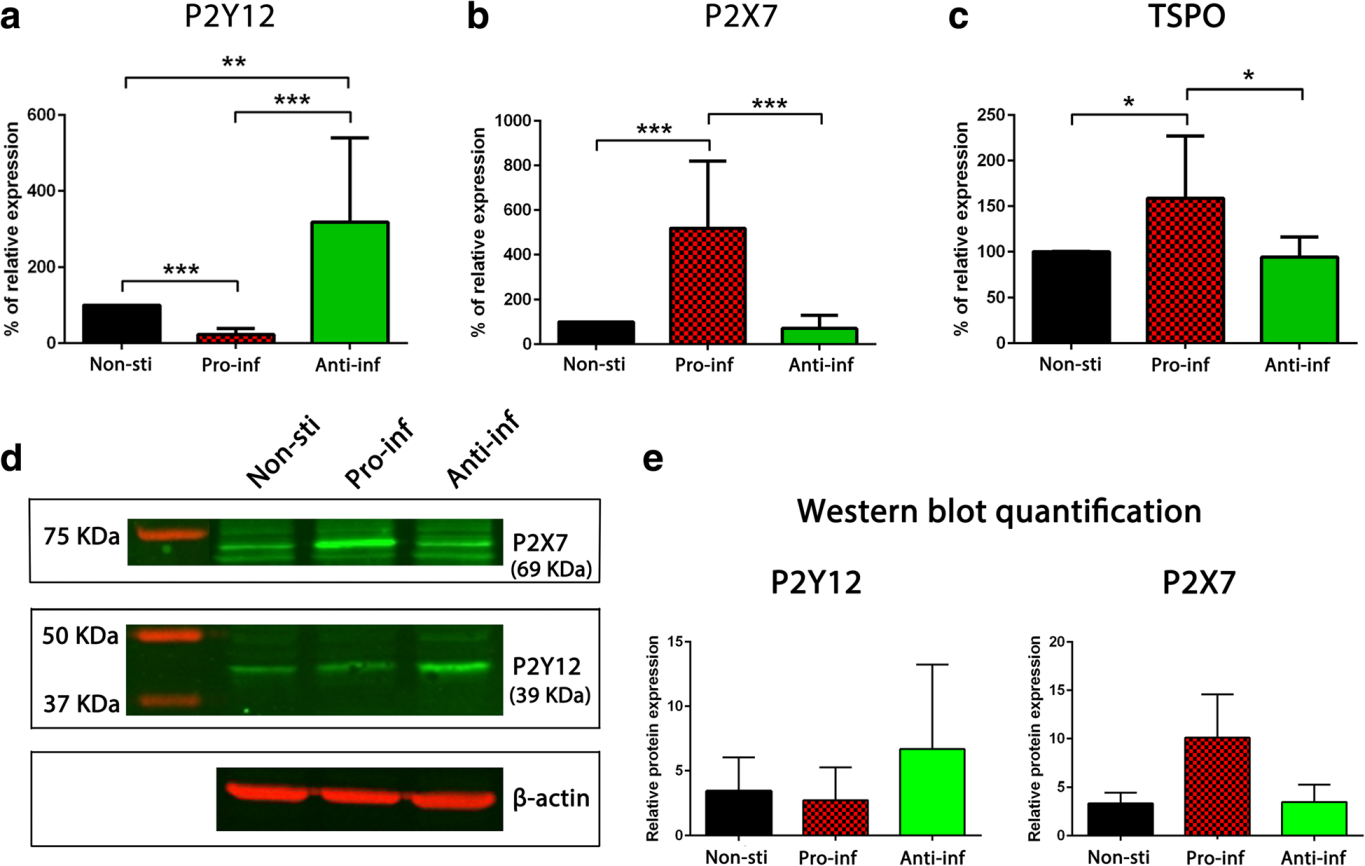
P2Y12R, P2X7R, and TSPO expression in non-stimulated (non-sti), pro-inflammatory (pro-inf), and anti-inflammatory (anti-inf) polarized human adult microglia. Quantitative PCR analysis and western blot were performed on mRNA and cell lysate collected from non-stimulated, LPS-stimulated pro-inflammatory, and IL-4-stimulated anti-inflammatory human cultured microglia. Expression level of P2Y12R (**a**), P2X7R (**b**), and TSPO (**c**) was evaluated. P2X7R and TSPO were significantly upregulated in pro-inflammatory microglia, and P2Y12R was significantly upregulated in anti-inflammatory microglia (*n* = 10 donors; each sample is done in duplicate). Representative western blot (**d**) and quantification (**e**) of P2Y12R and P2X7R protein expression in non-stimulated, pro-inflammatory, and anti-inflammatory human microglia (*n* = 2 donors). Error bars represent SD. Each sample was done in duplicate. (one asterisk) *p* < 0.05, (two asterisks) *p* < 0.01, (three asterisks) *p* < 0.001

To investigate the regulation of P2Y12R and P2X7R expression, human microglia were freshly isolated from 10 different adult post-mortem brains, cultured for 7 days and then polarized into an inflammatory phenotype with IFN-γ/LPS or anti-inflammatory phenotype with IL-4 as described previously [3] (Additional file 1: Figure S1). Expression levels of P2Y12R, P2X7R, and TSPO were measured using quantitative PCR. P2Y12R was significantly downregulated in inflammatory polarized microglia compared to non-stimulated microglia (fourfolds lower, *p* < 0.001). In anti-inflammatory polarized microglia, the expression of P2Y12R was strongly increased compared to non-stimulated microglia (threefolds higher, *p* < 0.01) and pro-inflammatory polarized microglia (12-folds higher, *p* < 0.001) (Fig. 1a).

P2X7R expression revealed an opposite expression pattern with a significant increase in pro-inflammatory microglia (fivefolds higher, *p* < 0.01) compared to non-stimulated microglia and anti-inflammatory stimulated microglia (fivefolds higher, *p* < 0.01). No significant change was observed in anti-inflammatory microglia, which indicates a strong association of P2X7R with a pro-inflammatory microglia phenotype (Fig. 1b). The increased expression of P2X7R in pro-inflammatory microglia and P2Y12R in anti-inflammatory microglia was also observed at the protein level as shown by western blot from the same donors (Fig. 1d, e). Protein level was only evaluated in two donors due to the low amount of microglia cells obtained from the isolations which makes it challenging to have enough protein for western blot analysis. We next investigated the expression of TSPO as this marker is considered to be the state of art for PET imaging of activated microglia. TSPO was highly expressed in pro-inflammatory microglia (1.5-folds higher, *p* < 0.05), albeit less pronounced compared to P2X7R (1.5-folds vs fivefolds, respectively). In anti-inflammatory microglia, no significant change of TSPO expression was observed compared to non-stimulated microglia (Fig. 1c).

### Expression of P2Y12R and P2X7R in multiple sclerosis lesions

**Fig. 2:**
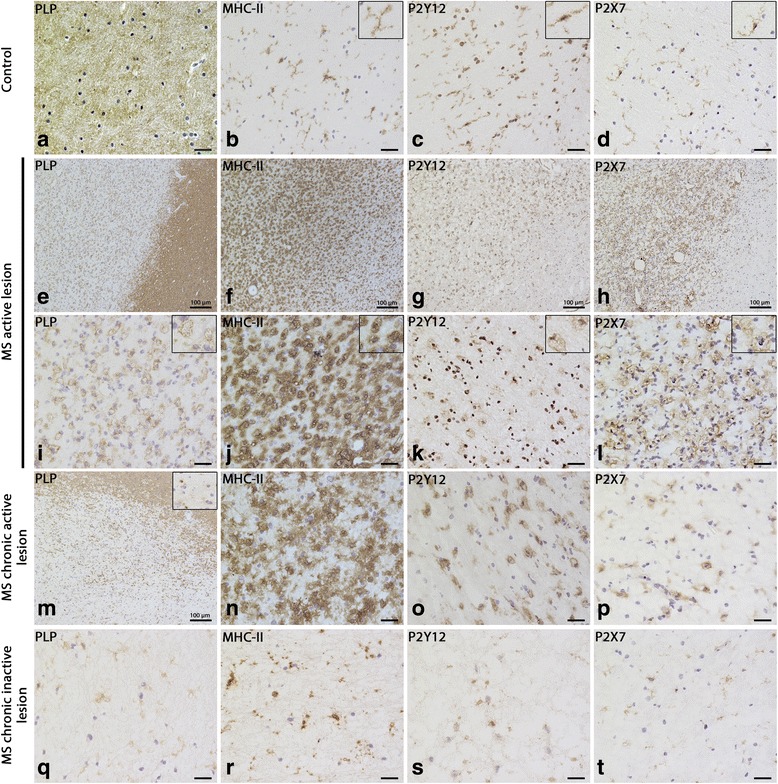
Expression of P2Y12R and P2X7R in controls, MS active, chronic active, and inactive lesions. PLP and MHC-II staining in different MS lesions and control (**a**, **b**, **e**, **f**, **i**, **j**, **m**, **n**, **q**, **r**). P2Y12R and P2X7R expression on ramified microglia in control human brain tissue (**c**, **d**). P2Y12R expression in MS active lesion (**g**, **k**), chronic active lesion (**o**), and chronic inactive lesion (**s**). P2X7R expression in MS active lesion (**h**, **l**), chronic active lesion (**p**), and chronic inactive lesion (**t**). The images were taken at the middle of the lesion for active sections and at the rim of the lesion for chronic active sections. Scale bar is 20 μm when not specified

**Fig. 3:**
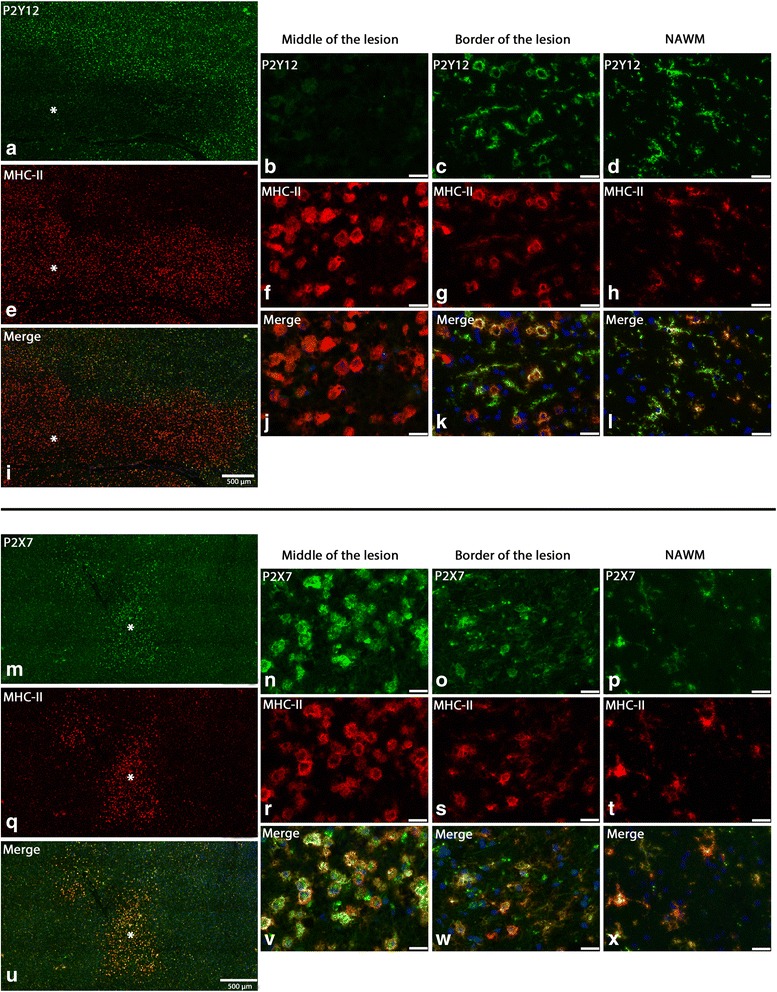
Expression of P2Y12R and P2X7R on MHC-II-positive cells in MS active lesion. Low-zoom images showing P2Y12R and MHC-II double staining in MS active lesion (**a**, **e**, **i**); asterisk indicates the center of the lesion. High-magnification images of P2Y12R and MHC-II double staining taken at the center of the lesion (**b**, **f**, **j**), the border of the lesion (**c**, **g**, **k**), and the NAWM (**d**, **h**, **l**). Low-zoom images showing P2X7R and MHC-II double staining in MS active lesion (**m**, **q**, **u**). High-magnification images of P2X7R and MHC-II double staining taken at the center of the lesion (**n**, **r**, **v**), the border of the lesion (**o**, **s**, **w**), and the NAWM (**p**, **t**, **x**). All images were acquired in the same session using same exposure time between different areas to allow comparison. Blue is nuclear staining with Hoechst. Scale bar is 25 μm when not specified

In order to investigate P2Y12R and P2X7R as targets for imaging microglia phenotype in MS, we analyzed their expression in white matter samples from three non-neurological controls, three active, two chronic active, and three chronic inactive MS lesions (control, Fig. 2a–d; active, Fig. 2e–l; chronic active, Fig. 2m–p, chronic inactive, Fig. 2q–t; Additional file 1: Figure S2-5). P2X7R and P2Y12R were expressed on ramified cells in control brain and normal-appearing white matter (NAWM) that were identified as microglia by their colocalization with MHC-II (Fig. 3). In inflamed MS lesions, P2X7R and P2Y12R are expressed on activated microglia characterized by their round shape and high MHC-II expression (Figs. 2 and 3; Additional file 1: Figure S2-S5). GFAP- (marking activated astrocytes) and CD31- (marking endothelial cells) positive cells in MS lesion, NAWM, or control tissue did not express P2X7R or P2Y12R (Additional file 1: Figure S7-10).

P2X7R and P2Y12R expression on microglia is affected by their activated state. To compare the change of expression of P2X7R and P2Y12R between ramified and activated microglia, we performed colocalization studies with MHC-II in active and chronic active MS lesions. P2X7R was more robustly expressed on activated microglia in active lesions and on microglia in the active rim of the chronic active lesions compared to microglia in the NAWM (Fig. 3m–x; Additional file 1: Figure S11). In contrast, P2Y12R immunolabeling showed a gradual decrease of expression on microglia from the NAWM towards the center of the active lesion (Fig. 3a–l). Interestingly, at the border of the active lesion, we observe a mixed population of activated microglia expressing either high or low levels of P2Y12R; the same pattern was also observed in activated microglia in the rim of the chronic active lesions (Additional file 1: Figure S12).

### P2Y12R and P2X7R expression on microglia in human MS lesions

**Fig. 4:**
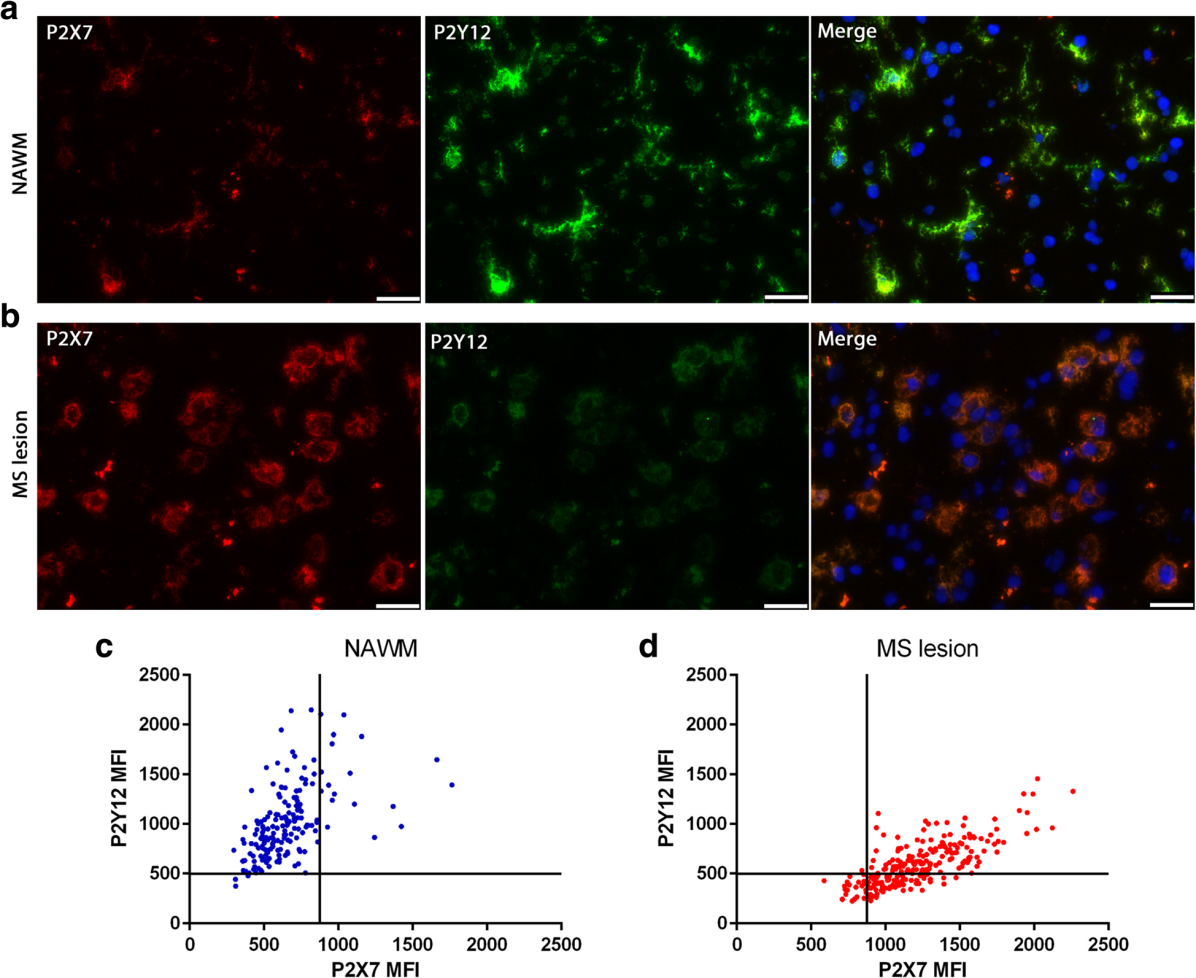
Expression of P2Y12R and P2X7R on microglia in NAWM and human MS active lesion. Double staining showing the expression of P2Y12R and P2X7R in NAWM (**a**) and MS active lesion (**b**). Images were collected from the same section with the same exposure time between different areas to allow comparison. Scatter plot showing quantification of P2Y12R and P2X7R fluorescence mean intensity (MFI) on microglia in NAWM (179 cells quantified) (**c**), and active MS lesion (241 cells quantified) (**d**). Each dot represents a single cell. All images for quantification were collected from a single section and were acquired in a single imaging session with the same exposure time. Regions of interest (ROIs) were drawn manually delineating each cell using Leica LAS AF software

We observe that P2X7R and P2Y12R are differentially expressed within MS active lesions. In order to investigate the dynamic of expression of P2X7R and P2Y12R, we compared their respective change of expression on ramified microglia in the NAWM and activated microglia in MS lesions. Enhanced expression of P2X7R on activated microglia in the MS lesion is accompanied by a decrease in microglial P2Y12R expression on the same cells (Fig. 4a, b). To further evaluate the relation between the expression of P2Y12R and P2X7R, we quantified their expression by drawing regions of interest (ROIs) around microglia in the NAWM and MS lesion. When comparing the microglia at the ramified less activated state to the highly activated state, we detected that in the majority of microglia an increase of expression of P2X7R coincides with a decrease of P2Y12R expression (Fig. 4c, d). We also observed a small population of activated microglia that shows an increase of P2X7R expression and no decrease in P2Y12R expression; these cells were mostly located at the border of the lesion.

### Expression of P2Y12R and P2X7R in acute EAE lesions

To investigate whether the change of expression of P2Y12R and P2X7R that we observed in post-mortem human MS tissue also occurs in EAE, a well-established animal model of MS, we performed immunohistochemistry on tissues from acute EAE induced in Lewis rats. We used cerebellum and brain stem tissue sections where the majority of neuroinflammation and immune cell infiltration occurs in this particular model. Tissues were collected from EAE animals at the peak of the disease (day 14 post-immunization) and in the recovery phase (day 20 post-immunization). Tissues from animals immunized with complete Freund’s adjuvant (CFA) were used as control. Microglia were stained with anti-CD11b, and sections were analyzed by differentiating a highly inflamed and a less inflamed area based on the CD11b staining.

We first investigated the expression of P2X7R and P2Y12R in control animals. P2Y12R immunoreactivity localized with CD11b-positive microglia in control brain tissue (Additional file 1: Figure S13). No P2X7R expression was detected in control brain tissue, which may be due to the low expression of P2X7R and/or the low sensitivity of the antibody (Additional file 1: Figure S16).

**Fig. 5:**
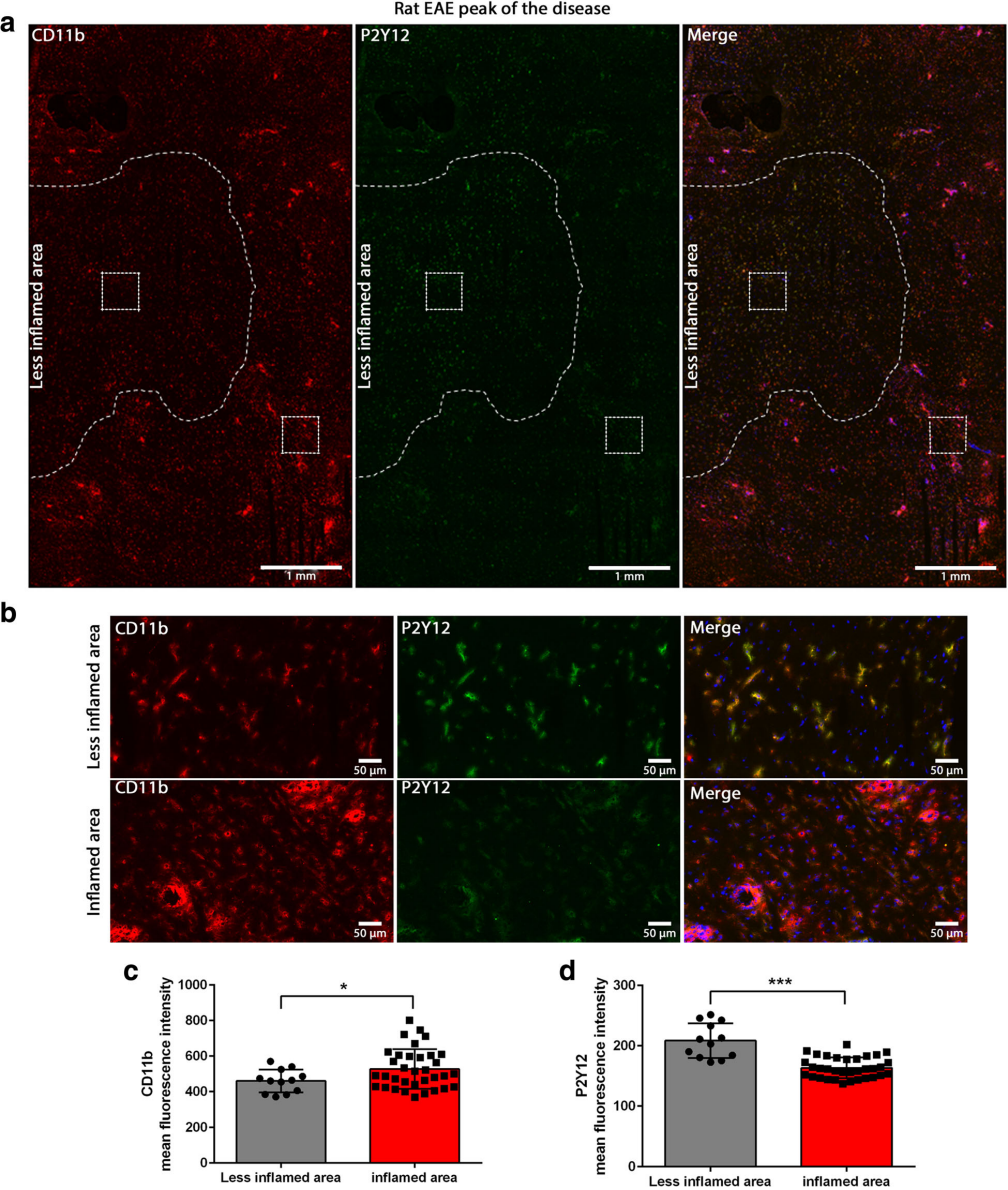
P2Y12R expression is downregulated on activated microglia in EAE at the peak of the disease. P2Y12R and CD11b double staining on EAE tissues from the peak of the disease showing a highly infiltrated and inflamed area, and a less inflamed area (**a**). Higher magnification images of P2Y12R and CD11b staining showing a reduction of P2Y12R expression on microglia in the high inflamed area compared the less inflamed area (**b**). Fluorescence quantification of CD11b (**c**) and P2Y12R (**d**) showing a significant increase in CD11b expression and a significant decrease of P2Y12R expression in the highly inflamed area compared to the less inflamed area. Each circle and square represents one drawn region of interest (ROI) and represents tissues from two different rats. Different groups were compared using the Student *t* test, (one asterisk) *p* < 0.05, (three asterisks) *p* < 0.0001. Blue is nuclear staining with DAPI

**Fig. 6:**
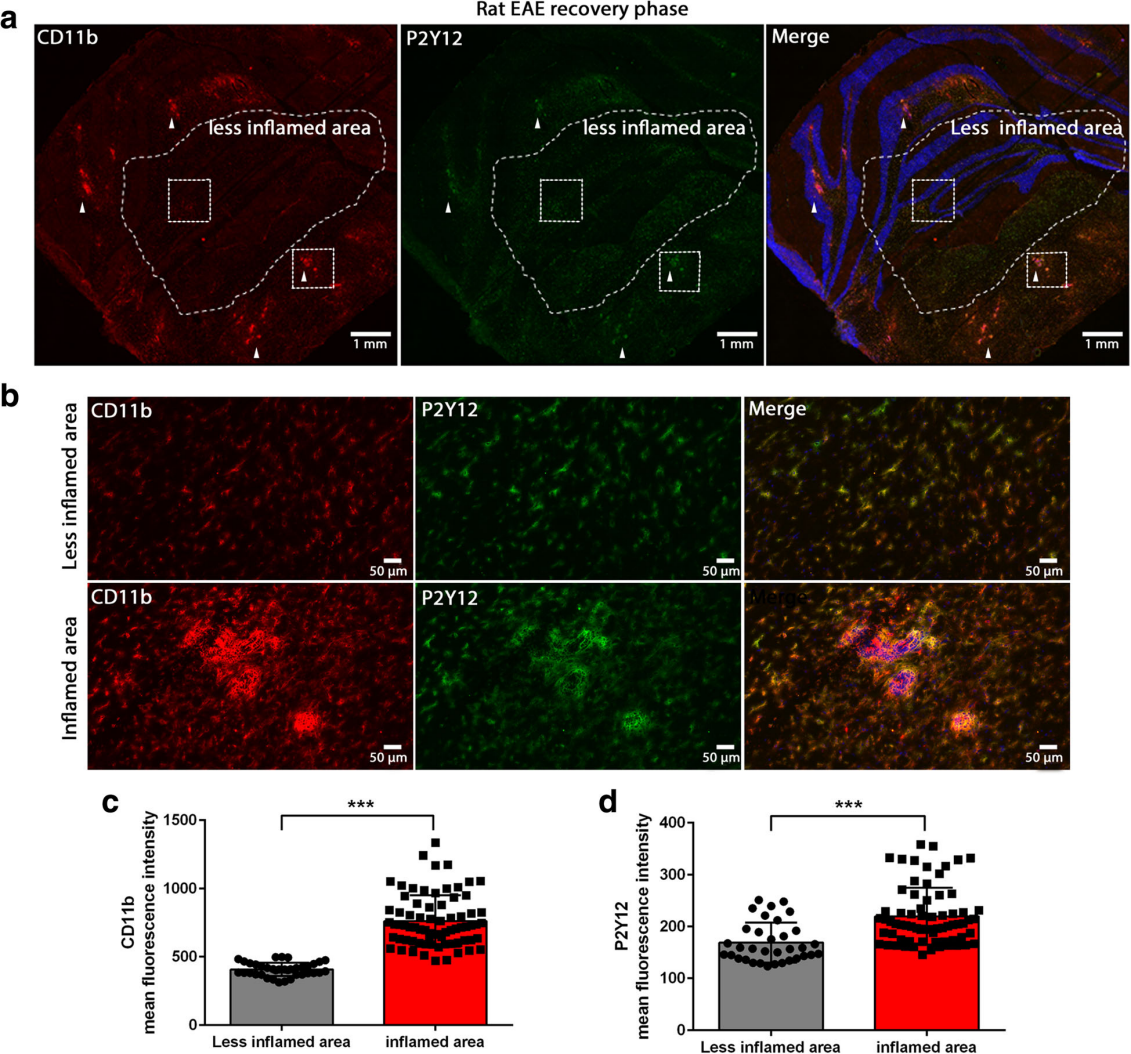
P2Y12R expression is upregulated on activated microglia in EAE in the recovery phase. P2Y12R and CD11b double staining on EAE tissues from the recovery phase showing a highly infiltrated and inflamed area, and a less inflamed area. Arrow head shows high P2Y12R staining on CD11b positive cells (**a**). Higher magnification images of P2Y12R and CD11b staining showing an increase of P2Y12R expression on microglia in the high inflamed area compared the less inflamed area (**b**). Fluorescence quantification of CD11b (**c**) and P2Y12R (**d**) showing a significant increase in CD11b and P2Y12R expression in the highly inflamed area compared to the less inflamed area. Each circle and square represents one drawn region of interest (ROI) and represents tissues from three different rats. Different groups were compared using student t-test, (one asterisk) *p* < 0.05, (three asterisks) *p* < 0.0001. Blue is nuclear staining with DAPI

At the peak of the disease, we observed a marked decrease of P2Y12R on CD11b-positive cells in the highly inflamed area. Quantification of the P2Y12R fluorescence signal revealed a significant reduction of the staining in the inflamed area compared to the less inflamed area (Fig. 5a–d). In EAE, at the end of the recovery phase, P2Y12R is markedly expressed on CD11b-positive microglia compared to microglia in the less inflamed area (Fig. 6a, b). The quantification of the P2Y12R expression showed a significant increase of the P2Y12R expression in the inflamed area compared to the less inflamed area (Fig. 6c, d). Infiltrating monocyte-derived macrophages (ED1-positive) in the perivascular area did not express P2Y12R (Additional file 1: Figure S14).

**Fig. 7:**
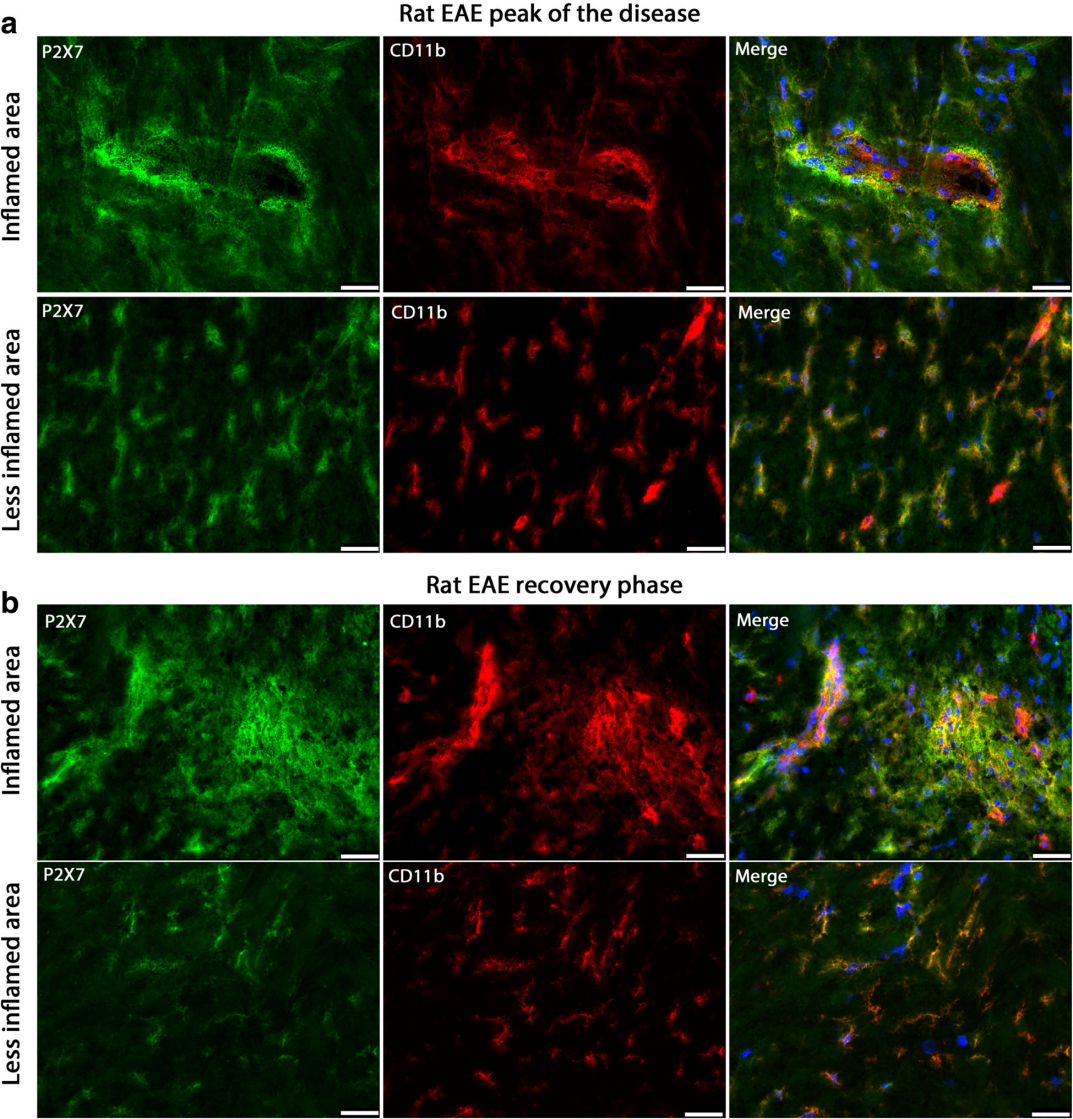
P2X7R expression in rat EAE at the peak of the disease and in the recovery phase. P2X7R and CD11b double staining on EAE tissue from the peak of the disease (**a**) and the recovery phase (**b**). Blue is nuclear staining with DAPI. Scale bar is 25 μm

P2X7R was highly expressed on CD11b-positive cells in the EAE tissue at the peak of the disease and at the end of the recovery phase. The expression of P2X7R on activated microglia in the inflamed area was higher compared to the ramified microglia in the less inflamed area (Fig. 7a, b).

### Binding of P2Y12R and P2X7R radiolabeled tracers on MS and acute EAE tissue

**Fig. 8:**
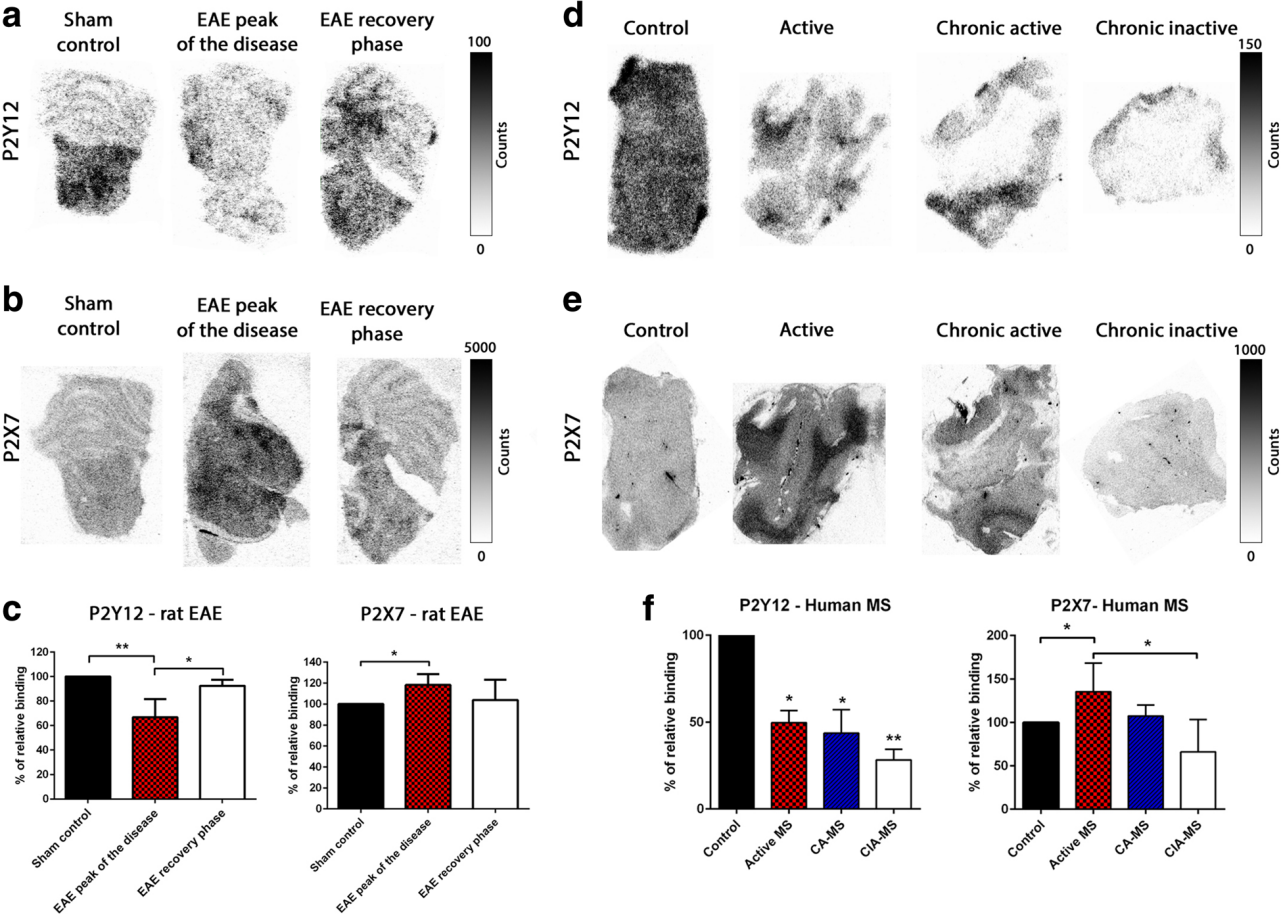
Autoradiography on acute rat EAE tissue and human MS tissue using P2Y12R and P2X7R radiotracers. Representative autoradiograms of carbon-11-labeled P2Y12R-ant on rat control and EAE tissues (**a**). Representative autoradiograms of tritium-labeled A-740003 (P2X7R specific antagonist) on rat control and EAE tissues (**b**). Quantification of binding of [^11^C]P2Y12R-ant and [^3^H]A-740003 on rat control and EAE tissues (data presented is the summary of *n* = 3 animals per time point and three independent experiments) (**c**). Representative autoradiograms of [^11^C]P2Y12R-ant on different human MS lesion types (**d**). Representative autoradiograms of tritium-labeled A-740003 (P2X7R specific antagonist) on different human MS lesion types (**e**). Quantification of binding of [^11^C]P2Y12R and [^3^H]A-740003 on different human MS lesions (data presented is a summary of three independent experiments using three non-neurological controls, three active, two chronic active, and three chronic inactive MS lesions) (**f**)

To investigate the binding of PET tracers and their ability to predict and follow the change of P2Y12R and P2X7R expression, we performed autoradiography binding experiment using a carbon-11-labeled specific P2Y12R tracer ([^11^C]P2Y12R-ant) and tritium-labeled P2X7R-specific tracer ([^3^H]A-740003) for binding to EAE and human MS post-mortem tissues. For these binding experiments, we used sections from the same human MS and EAE tissues blocks as for the immunostaining experiments. In tissue from acute EAE animals, we observed a significant decrease of binding of the [^11^C]P2Y12R-ant tracer at the peak of the disease compared to the control that increases back in the recovery phase. P2X7R tracer, [^3^H]A-740003, showed a significant increase of binding to EAE tissues from the peak of the disease compared to control. The binding of [^3^H]A-740003 slightly decreased in the recovery phase (Fig. 8a–c). In human MS tissues, the binding of [^11^C]P2Y12R-ant tracer was significantly decreased in all types of MS lesions (active, chronic active, and chronic inactive) compared to control (Fig. 8d, f). The binding of P2X7R tracer [^3^H]A-740003 was significantly increased only in the MS active lesion while there was a slight non-significant increase in chronic active lesions and a decrease in the chronic inactive lesions compared to control (Fig. 8e, f). We also performed blocking studies and post-autoradiography immunostaining; our data showed that the binding of both tracers is specific and matches the antibody staining (Additional file 1: Figure S17-19). The binding of the P2X7R and P2Y12R tracers correlated with our immunohistochemistry results which further validate these receptors as potential targets for use in PET imaging of activated microglia.

## Discussion

Our data show that P2X7R and P2Y12R can be used for PET tracer development and have the potential to discriminate between pro- and anti-inflammatory microglia in vivo by PET imaging upon successful development of a PET tracer. This may help in obtaining insights into the role of microglia in disease progression and enabling the development of novel treatment strategies aimed at altering microglial phenotype in order to promote neuro-protection. The pathogenesis of MS is complex, and the role of microglia in the development and resolving of the disease is still poorly understood mainly due to the lack of tools to visualize such dynamics. In vitro, microglia can be polarized into pro-inflammatory or anti-inflammatory phenotype depending on stimulus, but in vivo, it has been hypothesized that the microglia activation is very dynamic and show rather a spectrum of activation phenotypes with pro- and anti-inflammatory characteristics on either end of this spectrum. PET imaging is a non-invasive technique and the method of choice for imaging neuroinflammation due to its high sensitivity and quantifiable images. Our data suggests that purinergic receptors P2X7R and P2Y12R could be of high value as PET imaging targets in order to identify a pro- or anti-inflammatory-balanced microglia environment that may suggests new insights into disease progression towards worsening or more resolving state.

P2X7R is markedly expressed on activated microglia and infiltrated macrophages in inflamed MS tissue compared to microglia in the NAWM. P2X7R might be activated by increased levels of ATP that are released during the inflammatory process and cellular damage that is prominent in MS active lesions [27]. P2X7R signaling is also involved in the activation of the inflammasome and the release of IL-1β by activated microglia that is a main inflammatory cytokine potentially playing a role in the MS pathogenesis [28]. The increase in P2X7R receptor in MS and EAE were reported previously using western blot analysis on whole brain homogenate [17, 29] and on microglia/macrophages cells in spinal cord of MS lesion [30]. Our data suggests that microglial P2X7R immunoreactivity correlated with a pro-inflammatory environment and ongoing neuroinflammation. We also observed an increase in P2X7R expression on activated microglia and macrophages at the peak of EAE when the level of inflammation is at the highest. Surprisingly, P2X7R is not reduced on activated microglia in the recovery phase of the EAE. Inflammatory cytokines, i.e., IL-17, IFN-γ, TNF-α, are still present in the recovery phase, but are counter balanced by enhanced levels of anti-inflammatory cytokines, i.e., IL-4 and IL-10 [31]. This implies that P2X7R expression on microglia is less regulated by anti-inflammatory stimuli that occur in the recovery phase in EAE and more responsive to inflammatory cytokines.

P2Y12R expression behaved in opposite direction to P2X7R where the expression was significantly downregulated on activated microglia in MS lesion compared to the microglia in the NAWM. In the EAE model at the peak of the disease, we observe a downregulation of P2Y12R expression on activated microglia in line with earlier reports [13, 22, 32]. However, in the recovery phase of EAE, P2Y12R expression was robustly increased on activated microglia suggesting that P2Y12R expression is more regulated by anti-inflammatory cytokines. In a recent study, Zrzavy et al. reported that a loss of P2Y12R expression on microglia was observed in both active MS lesions and the surrounding normal-appearing white matter (NAWM), suggesting that the chronic inflammatory environment in MS may contribute to the P2Y12R downregulation [32]. Moreover, a higher expression of P2Y12R was detected on microglia in inactive lesions where inflammation was subsided which support our observations about P2Y12R dynamic expression and sensitivity to cytokine environment.

It is apparent that P2X7R and P2Y12R are differentially expressed when comparing ramified microglia to activated microglia in MS lesions; an upregulation of P2X7R is associated with a downregulation of P2Y12R. Our in vitro data using human microglia polarized into pro- or anti-inflammatory phenotypes showed similar responses of P2X7R and P2Y12R expression and correlates with our observation in MS and EAE tissues. P2Y12R response to pro- or anti-inflammatory stimuli in isolated microglia was also in accordance with previous report by Moore et al. [33]. An upregulation of P2Y12R could occur in the remission phase of MS as it happened in the recovery phase of the EAE, but the limitation of post-mortem tissue make it challenging to investigate, which emphasize the importance of PET imaging for non-invasive evaluation of such molecular processes. In addition, the use of M1 and M2 markers to define pro-inflammatory vs anti-inflammatory microglia phenotypes has drawbacks as most of the microglia in the MS lesions adopt an intermediate M1/M2 phenotype [4]. This further stress the need of specific PET imaging markers for the visualization of pro- and anti-inflammatory microglia status in vivo.

Thorough examination of the immunostainings revealed a profound microglial staining pattern of P2Y12R expression. No staining was observed in other cell types, including astrocytes and endothelial cells. These results are in accordance with other studies showing that the expression of P2Y12R is restricted to microglia [21, 22, 32]. However, the specificity of P2X7R to microglia is more controversial. Our results show that P2X7R expression is restricted to microglia and macrophages in MS lesion and NAWM which is not fully in line with other studies showing its expression on other cells in the brain [34]. We cannot exclude the expression of P2X7R on other brain cells; however, the expression level may be lower compared to microglia and the differences in antibody used or tissue treatment could be the reasons of the discrepancy.

It is crucial that PET tracers are able to reflect the changes of expression for these two receptors that we observe in MS and EAE. Our autoradiography data on EAE and human MS tissues show that the binding of the radiolabeled P2X7R and P2Y12R antagonists are in strong accordance with the antibody staining strengthening the rationale of using PET imaging for evaluating the expression of these two receptors. Our autoradiography results give further support to P2X7R and P2Y12R as valuable targets for PET imaging of pro- and anti-inflammatory microglia and that the overall change of expression is significant enough to be detected by in vivo imaging. Combining PET imaging of these two receptors may also provide some insight into the pathological type of lesions in MS as we observe differential tracers binding in different types of MS lesions.

Altogether, our data shows that P2X7R and P2Y12R are PET targetable receptors that have the potential of discriminating between a pro- or anti-inflammatory microglia environment which may help in predicting a worsening or more resolving neuroinflammation in MS and EAE. A possible scenario is that an increase uptake of P2X7R tracer that is accompanied by a decrease uptake of P2Y12R may indicate a progressing MS lesion, while upon immunomodulatory treatment if an increase of P2Y12R uptake is observed, this may indicate that the lesion is shifting to more anti-inflammatory environment and thus indicating a possible resolution of neuroinflammation and treatment efficiency. To that end, we are currently finalizing the characterization of specific P2X7R and P2Y12R PET tracers for the next step of in vivo evaluation of microglia and/or macrophage dynamics in the EAE model. Moreover, a new P2X7R PET tracer was recently reported and showed promising results in an in vivo model in which the human P2X7 was overexpressed through an AAV vector [35]. In addition, the change of P2X7R and P2Y12R expression on activated microglia is not restricted to MS and EAE and has been also described in other neuroinflammatory disease.

## Conclusion

The data we present in this study is the first step in the process that may lead to better understanding of the role of microglia in MS and other neuroinflammatory diseases. Furthermore, it may contribute to a better understanding of the relation between microglia activation status and neurodegeneration, and in the development of new immunomodulatory and disease-modifying therapies.

## Additional file

Additional file 1: Includes supplementary figures and tables to support the data presented in the main manuscript. (DOCX 14306 kb)

## Abbreviations

CFA: Complete Freund’s adjuvant
CNS: Central nervous system
EAE: Experimental autoimmune encephalomyelitis
GM-CSF: Granulocyte-macrophage colony-stimulating factor
LSC: Liquid scintillation counting
MS: Multiple sclerosis
NAWM: Normal-appearing white matter
PET: Positron emission tomography
PLP: Proteolipid protein
QPCR: Quantitative real-time polymerase chain reaction
RCY: Radiochemical yield
ROIs: Regions of interest
TSPO: Translocator protein
