# Supplementary material for: Purinergic receptors P2Y12R and P2X7R: potential targets for PET imaging of microglia phenotypes in multiple sclerosis
Source: J Neuroinflammation. 2017 Dec 22;14:259. doi: 10.1186/s12974-017-1034-z (PMC5741931; doi:10.1186/s12974-017-1034-z)
Supplement: Additional file 1: — Includes supplementary figures and tables to support the data presented in the main manuscript. (DOCX 14306 kb) [file 12974_2017_1034_MOESM1_ESM.docx]

**Additional file 1:**


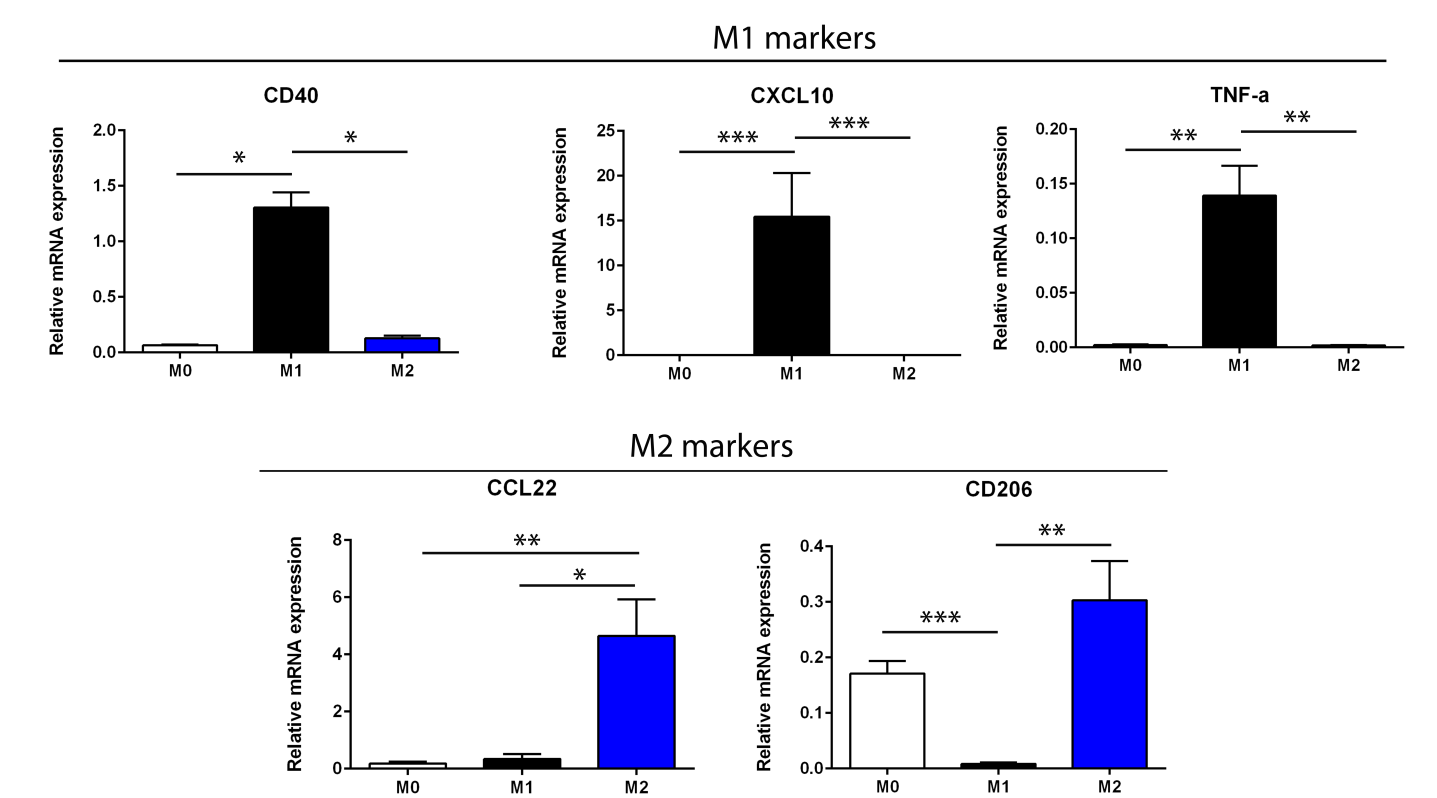


**Figure S1. Expression of M1 and M2 markers in human microglia after polarization with LPS/IFNγ (M1) or IL-4 (M2)**

Quantitative PCR analysis was performed on mRNA collected from unstimulated M0, LPS stimulated M1, and IL-4 stimulated M2 human cultured microglia. Expression level of CD40, CXCL10, TNFα, CCL22, and CD206 was evaluated. CD40, CXCL10, TNFα were significantly upregulated in M1 microglia. CCL22, and CD206 were significantly upregulated in M2 microglia. Error bars represent SD of five different donors. Each sample was done in duplicate. (*) p < 0.05, (**) p < 0.01, (***) p < 0.001.


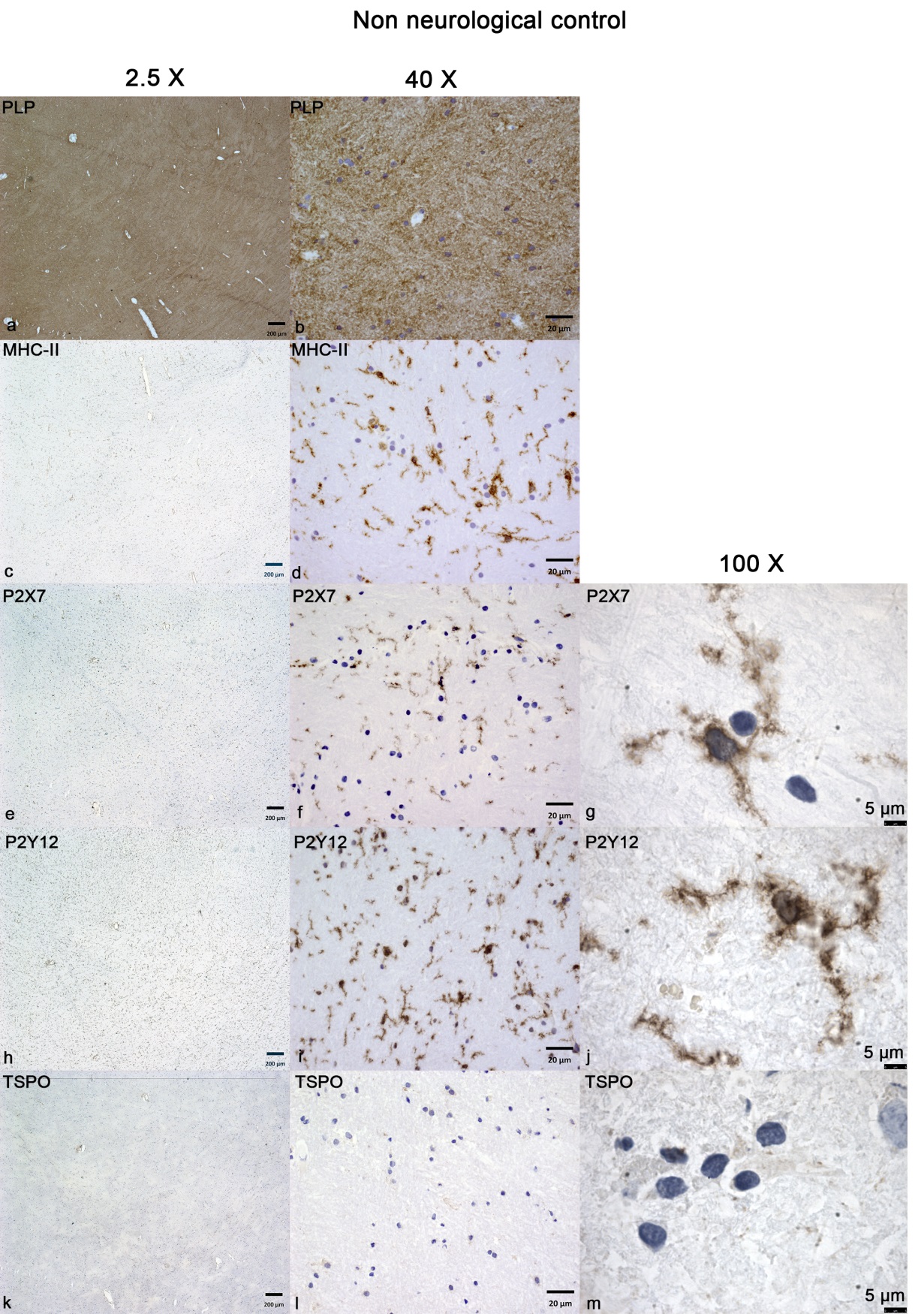


**Figure S2. Expression of P2X7R, P2Y12R, and TSPO in non-neurological control brain.** Staining of PLP (a, b), MHC-II (c, d), P2X7R (e, f, g), P2Y12R (h, I, j) and TSPO (k, l, m).


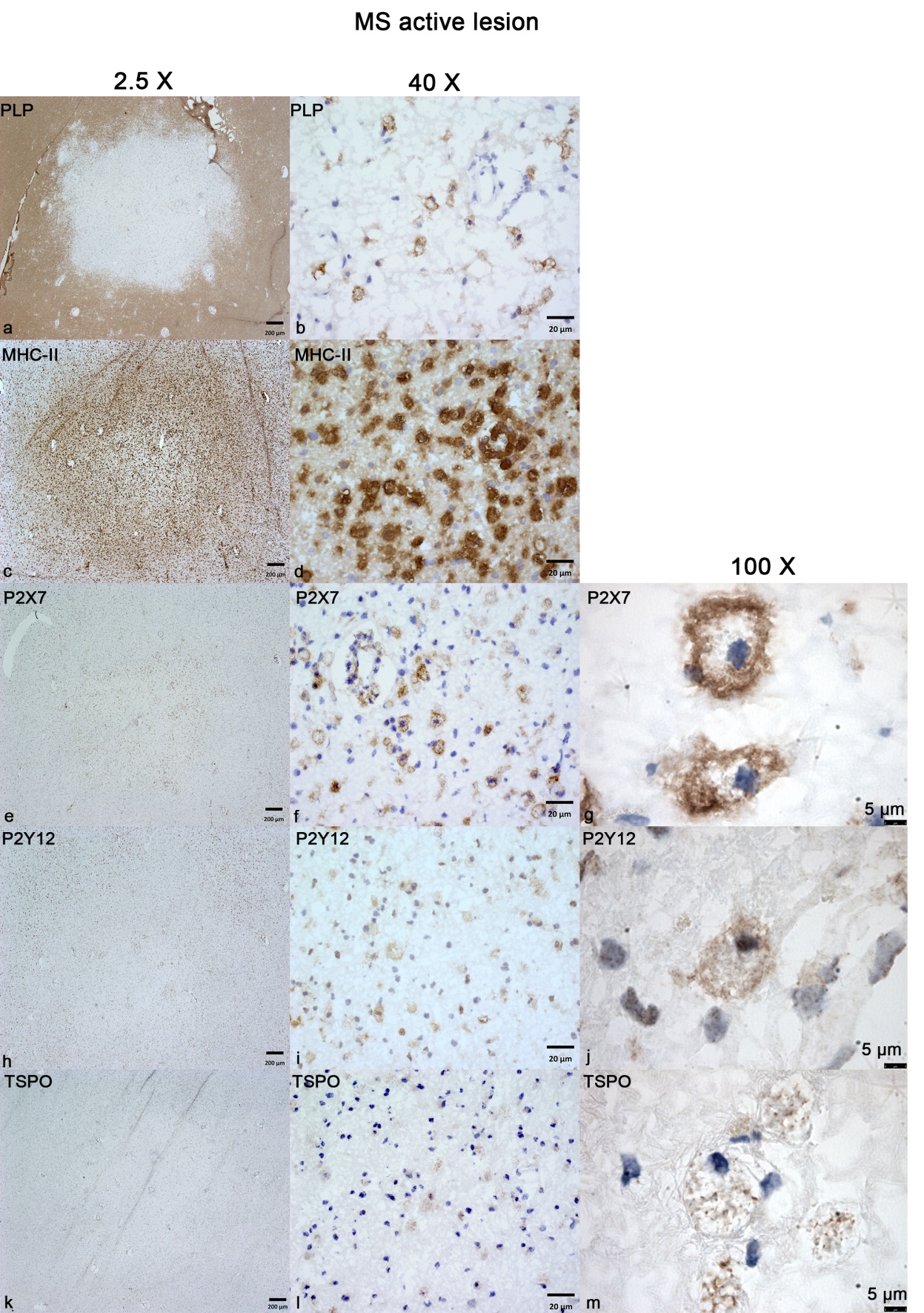


**Figure S3. Expression of P2X7R, P2Y12R, and TSPO in MS active lesion.** Staining PLP (a, b), MHC-II (c, d), P2X7R (e, f, g), P2Y12R (h, I, j) and TSPO (k, l, m).


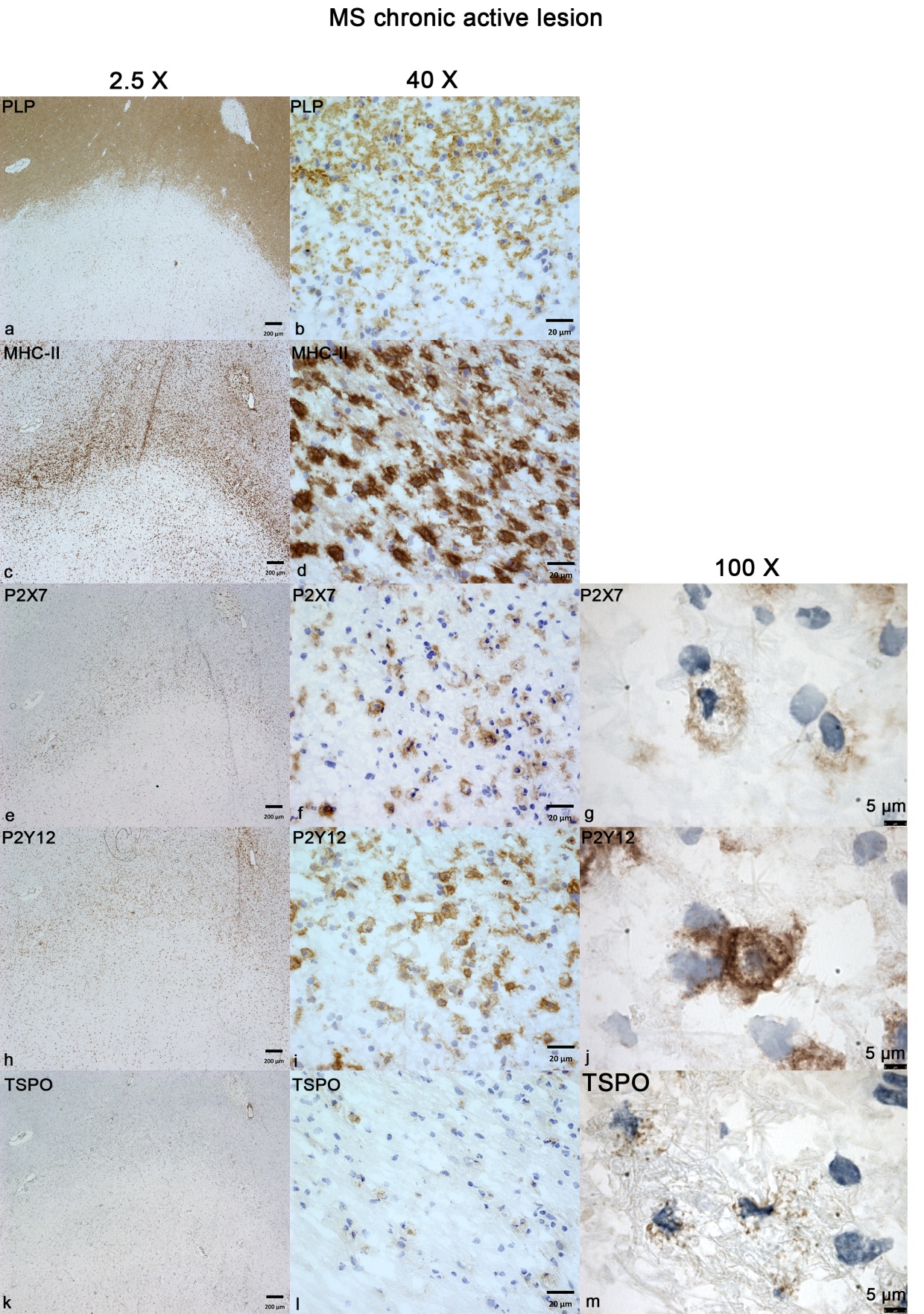


**Figure S4. Expression of P2X7R, P2Y12R, and TSPO in MS chronic active lesion.** Staining of PLP (a, b), MHC-II (c, d), P2X7R (e, f, g), P2Y12R (h, I, j) and TSPO (k, l, m).


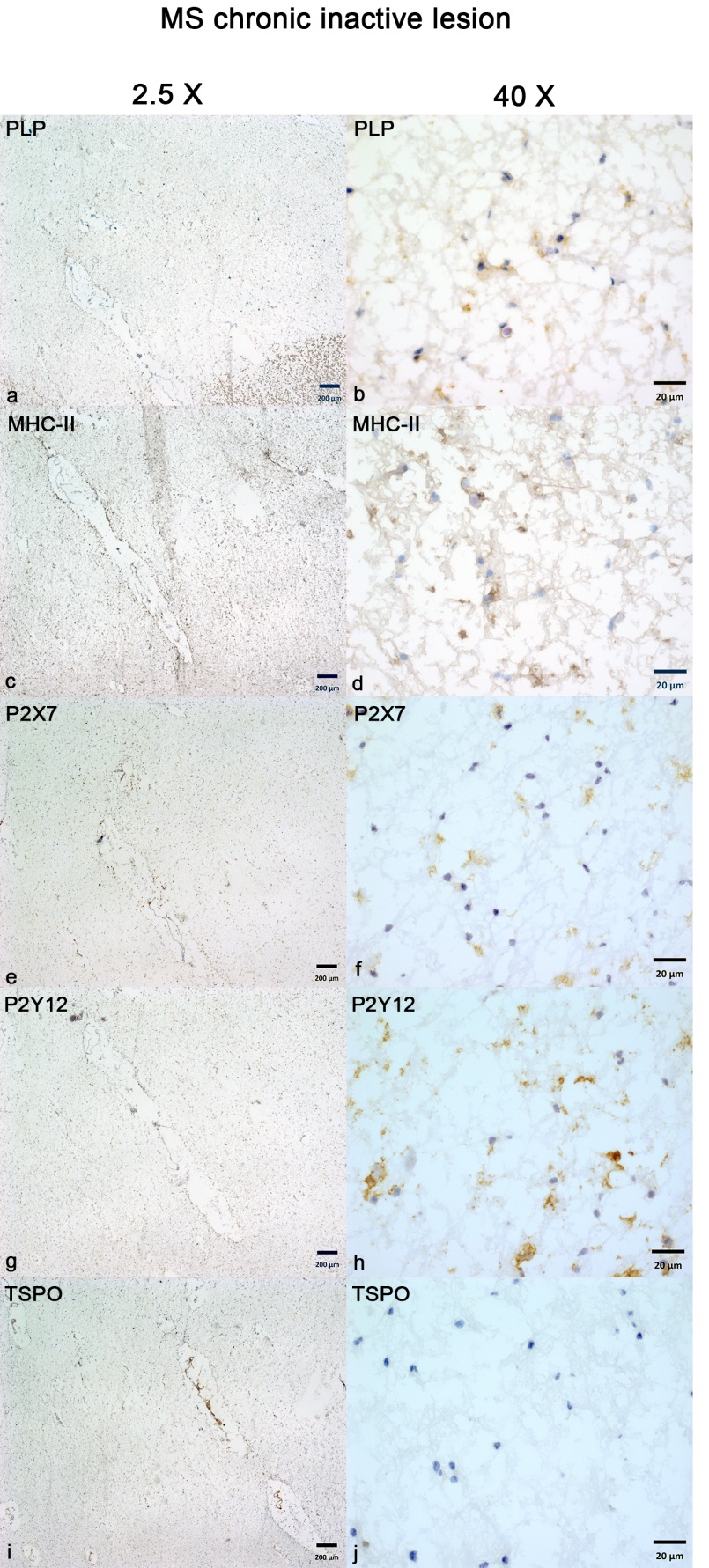


**Figure S5. Expression of P2X7R, P2Y12R, and TSPO in MS chronic inactive lesion.** Staining of PLP (a, b), MHC-II (c, d), P2X7R (e, f, g), P2Y12R (h, I, j) and TSPO (k, l, m).


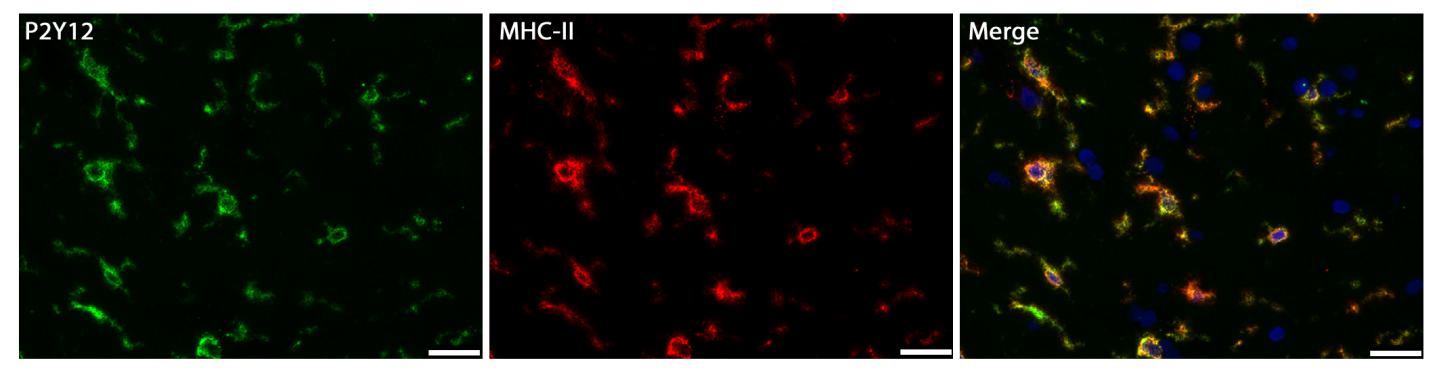


**Figure S6. Double staining of P2Y12R with MHC-II in normal control human brain.** We observe a complete colocalization between the P2Y12R and MHC-II staining. Blue is nuclear staining with DAPI. Scale bar 25 µm.


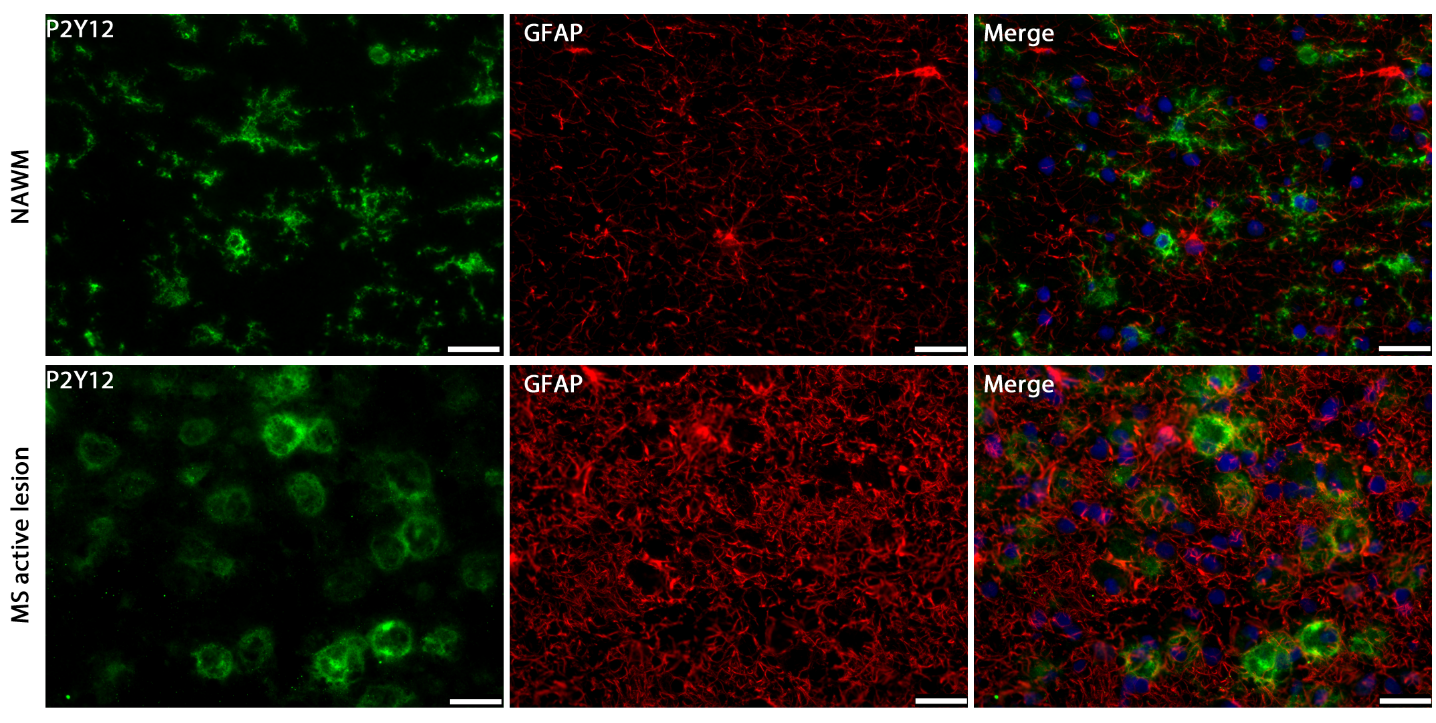


**Figure S7. P2Y12R expression on astrocytes in NAWM and MS active lesion.** No colocalization of P2Y12R and GFAP staining was found in NAWM and MS active lesion showing no expression of P2Y12R on astrocytes. Blue is nuclear staining with Hoechst. Scale bar 25 µm.


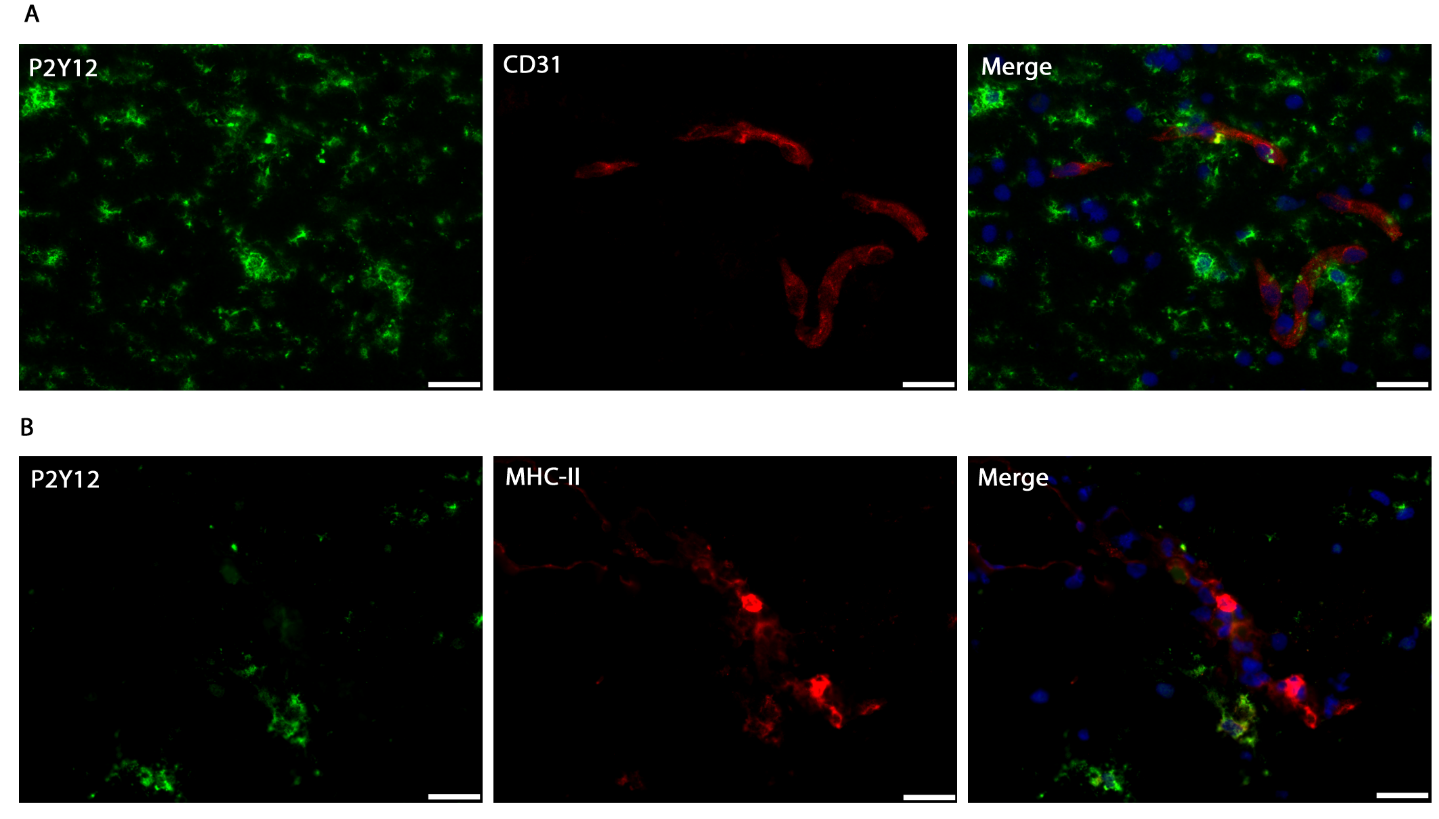


**Figure S8. P2Y12R expression on endothelial cells and perivascular macrophages in human brain.** P2Y12R staining does not colocalize with CD31 staining showing no expression of P2Y12R on endothelial cells (A). Perivascular macrophages stained with MHC-II does not express P2Y12R (B). Blue is nuclear staining with Hoechst. Scale bar 25 µm.


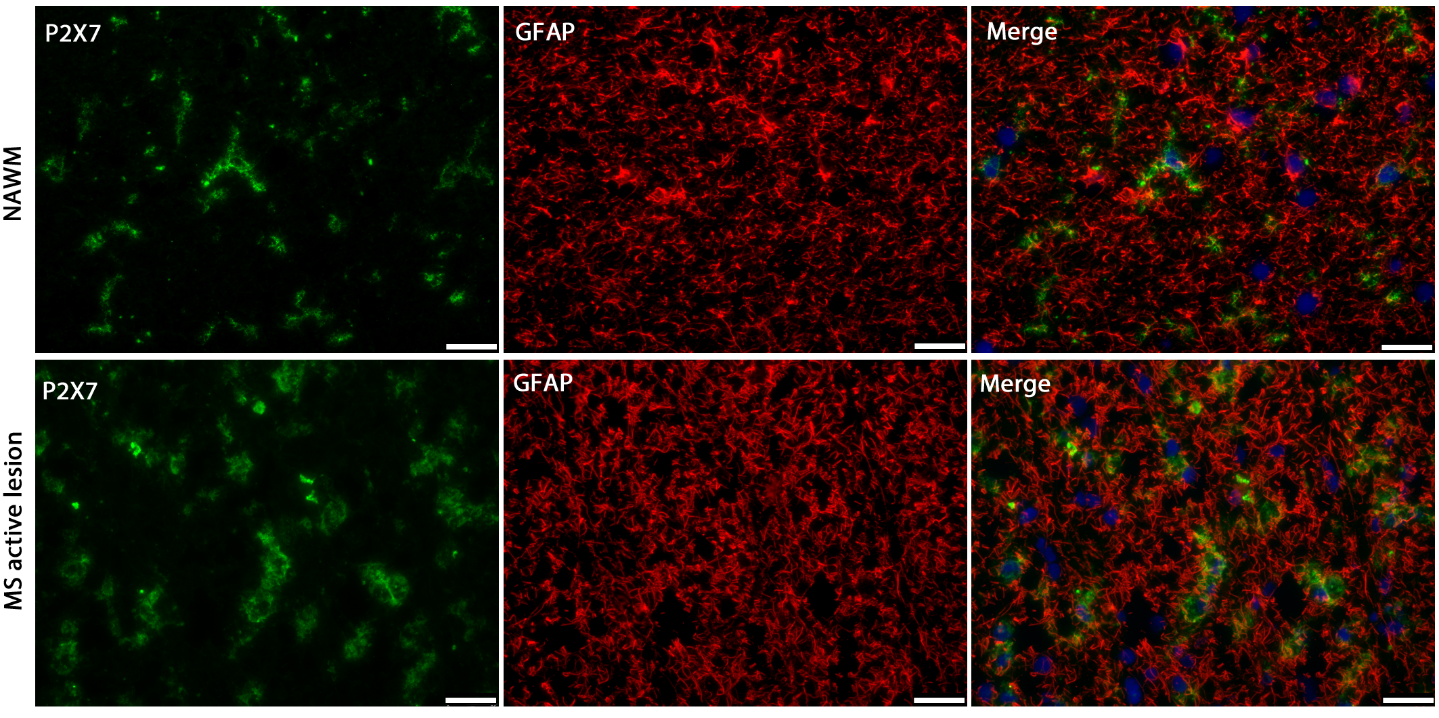


**Figure S9. P2X7R expression on astrocytes in NAWM and human MS active lesion.** No colocalization of P2X7R and GFAP staining in NAWM and MS active lesion showing no expression of P2X7R on human astrocytes. Blue is nuclear staining with Hoechst. Scale bar 25 µm.


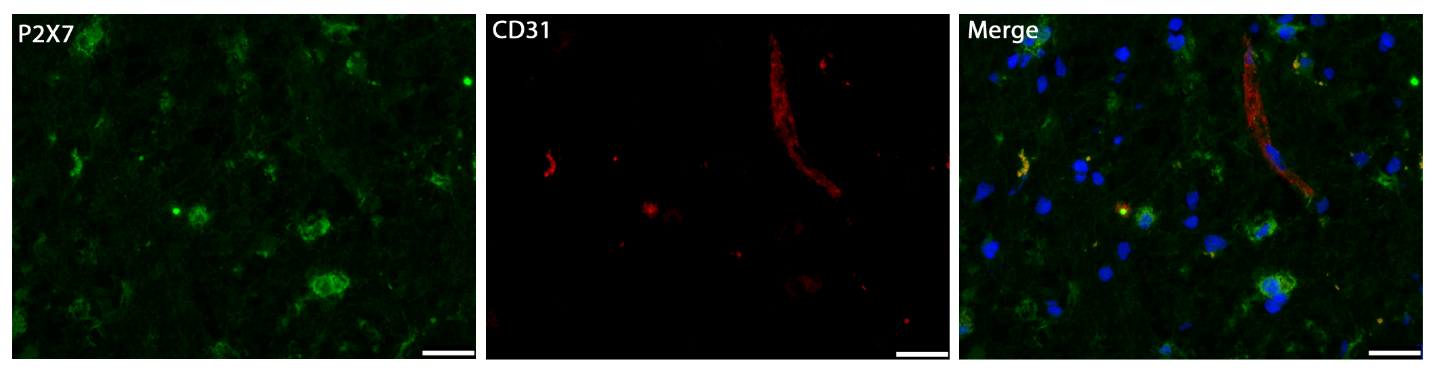


**Figure S10. P2X7R expression on endothelial cells in human MS brain.** P2X7R staining does not colocalize with CD31 staining showing no expression of P2X7R on endothelial cells. Blue is nuclear staining with Hoechst. Scale bar 25 µm.


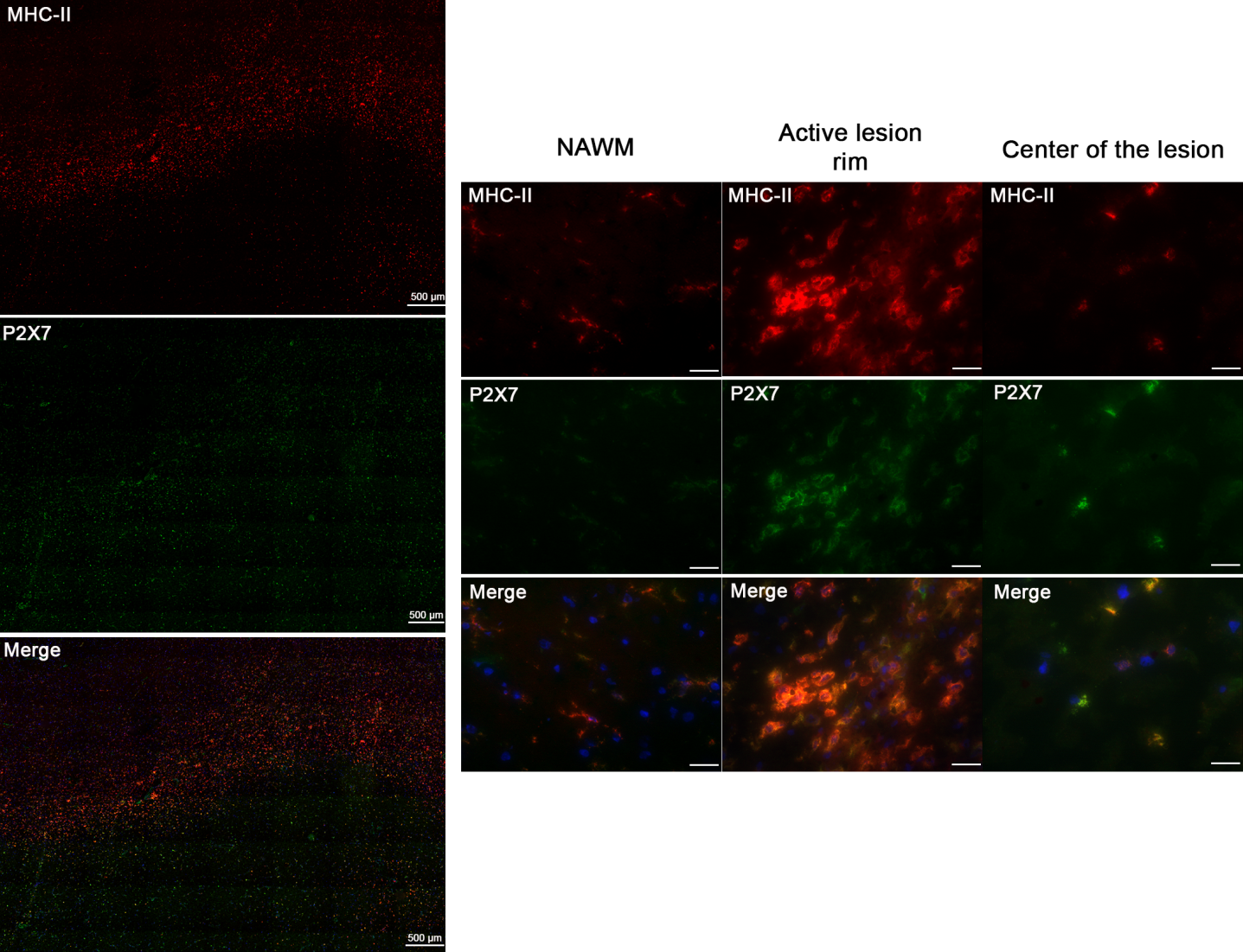


**Figure S11. Expression of P2X7R on MHC-II positive cells in MS chronic active lesion.** Low zoom images showing P2X7R and MHC-II double staining in MS chronic active lesion (left panel). High magnification images of P2X7R and MHC-II double staining taken at the NAWM, the active border of the lesion, and the center of the lesion. P2X7R expression is upregulated on activated microglia at the rim of the lesion compared to the ramified microglia in the NAWM. The few microglia present in the center of the lesion also express high level of P2X7R. All images were acquired on the same session using same exposure time between different areas to allow comparison. Blue is nuclear staining with Hoechst. Scale bar 25 µm when not specified.

**
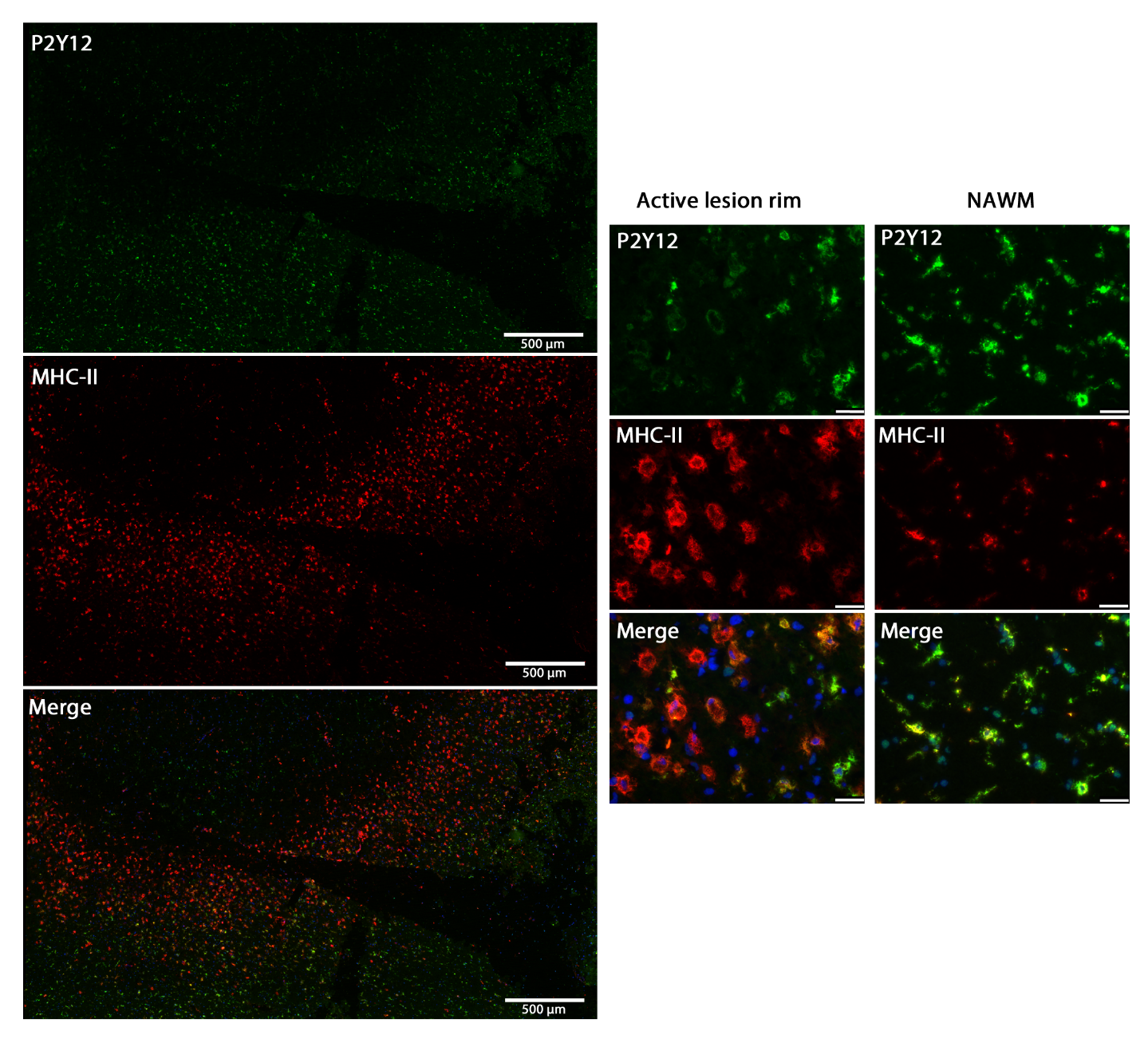
**

**Figure S12. Expression of P2Y12R on MHC-II positive cells in MS chronic active lesion.** Low zoom images showing P2Y12R and MHC-II double staining in MS chronic active lesion (left panel). High magnification images of P2Y12R and MHC-II double staining taken at the active border of the lesion, and the NAWM. We observe a mix of high and low P2Y12R expressing microglia at the rim of the lesion. All images were acquired on the same session using same exposure time between different areas to allow comparison. Blue is nuclear staining with Hoechst. Scale bar 25 µm when not specified.


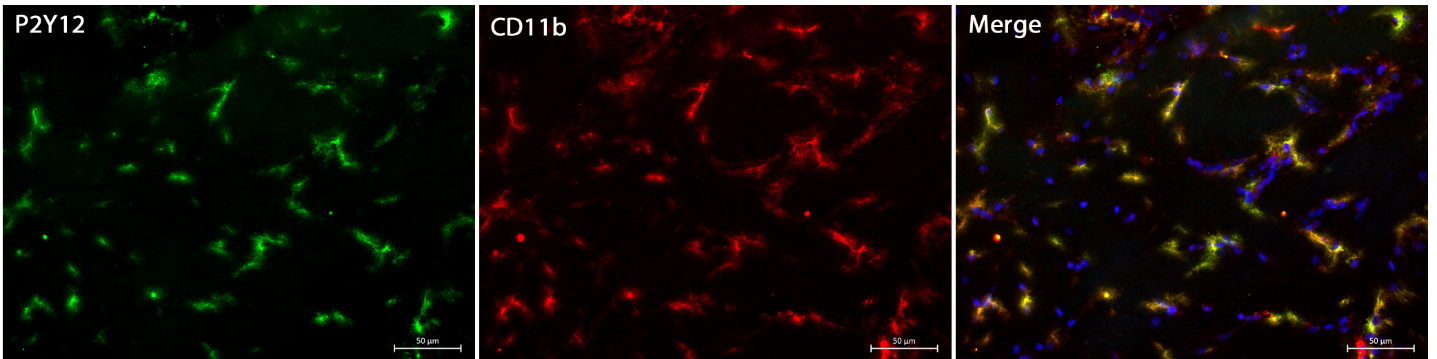


**Figure S13. P2Y12R expression on microglia in control rat brain.** Immunostaining with P2Y12R and CD11b in normal rat brain shows perfect colocalization between the two staining and indicating that only microglia expresses P2Y12R in rat brain. Blue is nuclear staining with DAPI.


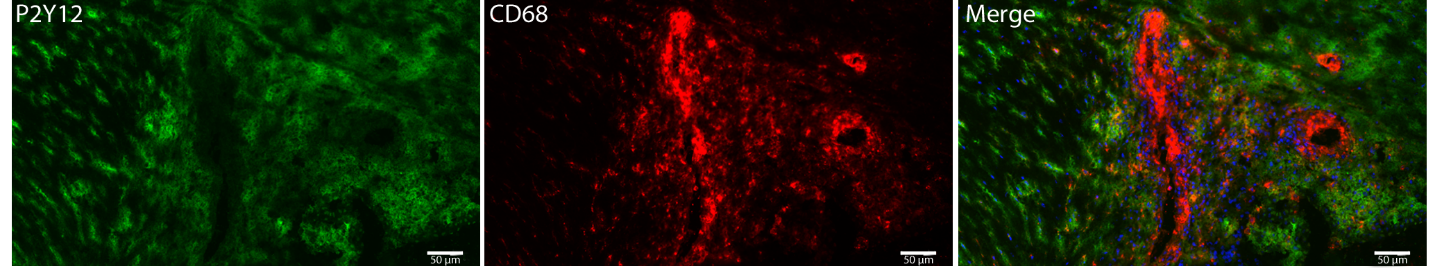


**Figure S14. Expression of P2Y12R on infiltrated macrophages in rat EAE in the recovery phase.** Perivascular infiltrated macrophages highly expressing ED1 does not express P2Y12R. Blue is nuclear staining with DAPI.


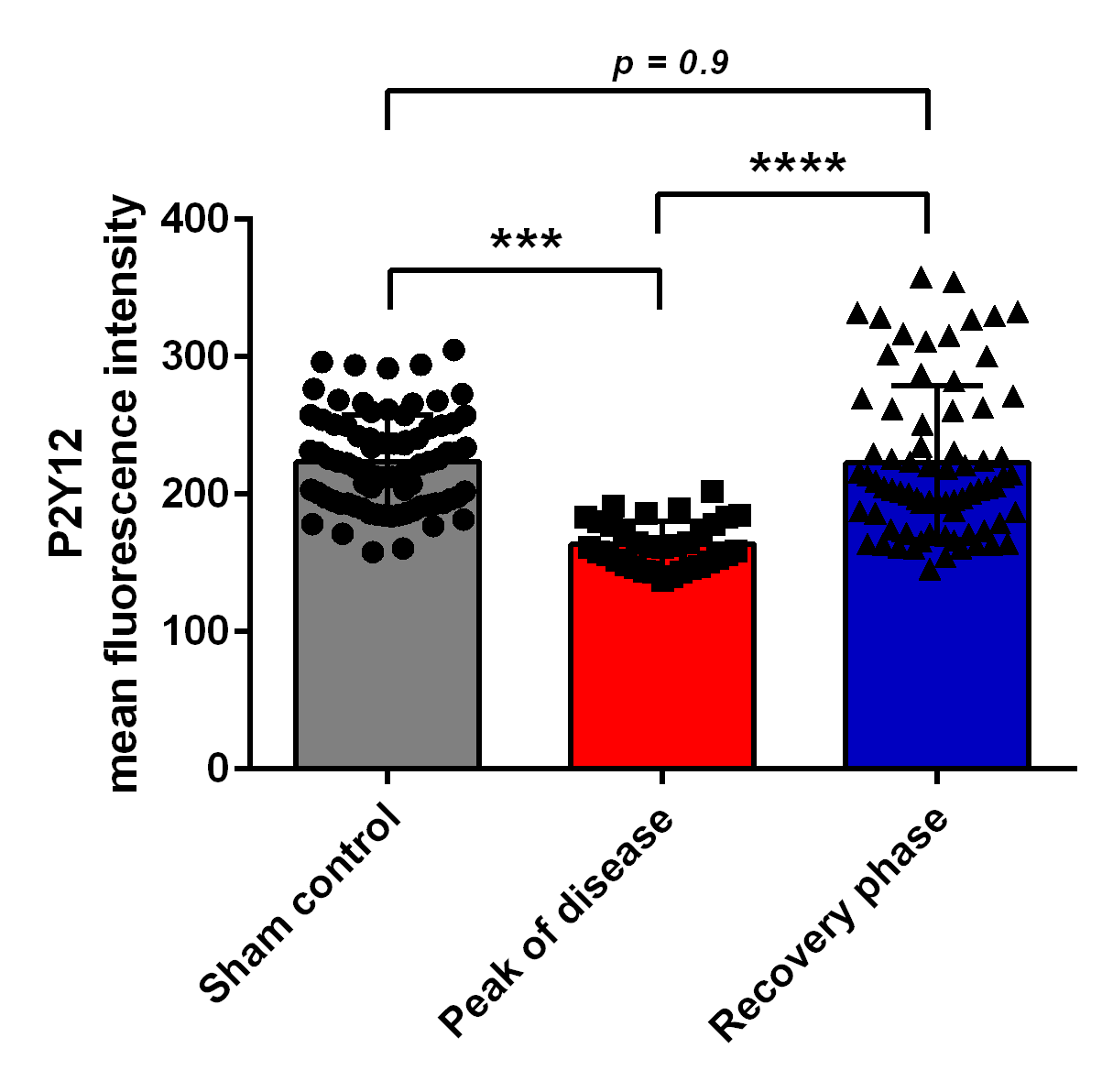


**Figure S15. Quantification of P2Y12R immunofluorescence from control rat brain sections and EAE at the peak of the disease and in the recovery phase.** We observe a significant reduction in the expression of P2Y12R in the EAE tissue at the peak of the disease compared to control. In the recovery phase the expression increases to the same level as control.

The staining was performed on tissue from control CFA immunized rats (n=3 different rats), and EAE rats sacrificed at the peak of the disease (at 14 dpi, n=2 different rats) and in the recovery phase (at 20 dpi, n=3 different rats). Staining of all tissue was performed in a single run. The whole slide was imaged and the images were acquired in a single session using the same exposure time between different sections. Each (●), (■) and (▲) represents one drawn region of interest (ROI). Different groups were compared using student t-test, (****) p < 0.0001.


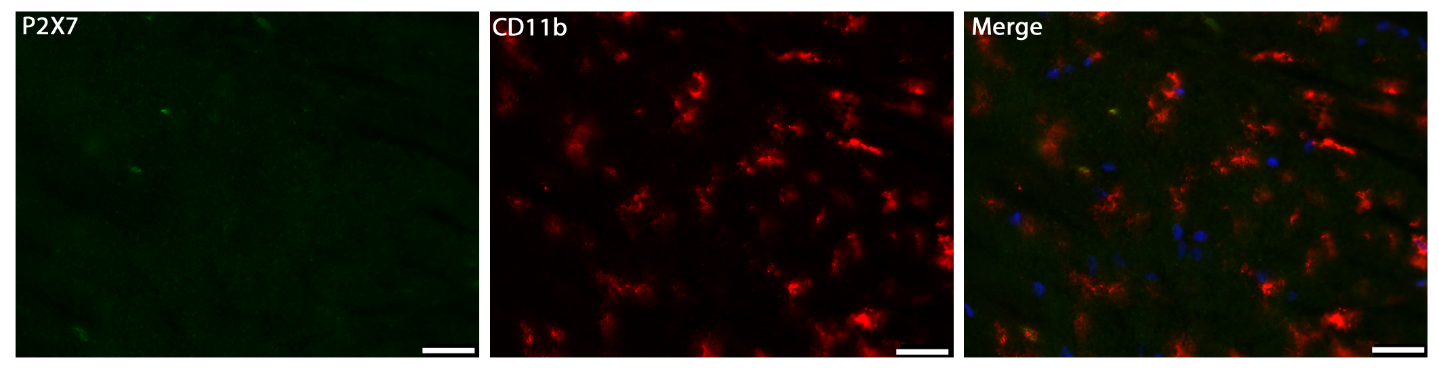


**Figure S16. P2X7R expression on microglia in control rat brain.** Double staining with P2X7R and CD11b in normal rat brain shows negative P2X7R staining of resting microglia. Blue is nuclear staining with DAPI. Scale bar 25 µm.


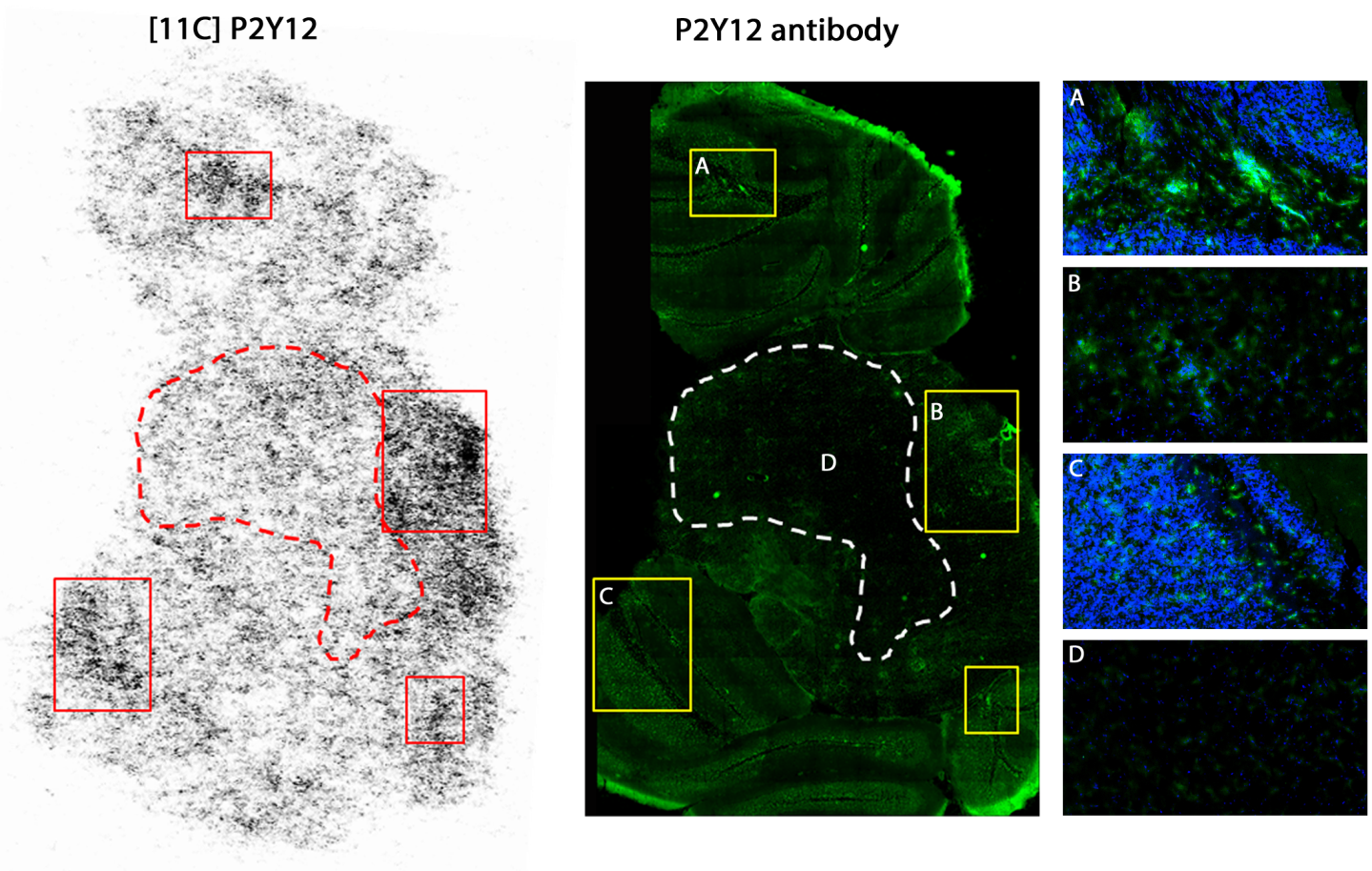


**Figure S17. Post [^11^C]P2Y12R-ant autoradiography immunostaining with P2Y12R antibody on rat EAE tissue.** After autoradiography experiment with [^11^C]P2Y12R-ant tracer, sections were fixed with acetone and immunostaining was performed using P2Y12R antibody according to the same staining protocol described in materials and methods. The high binding of the [^11^C]P2Y12R tracer colocalize with the high staining of the P2Y12R antibody (yellow boxes) observed by immunofluorescence. Blue is nuclear staining with DAPI.


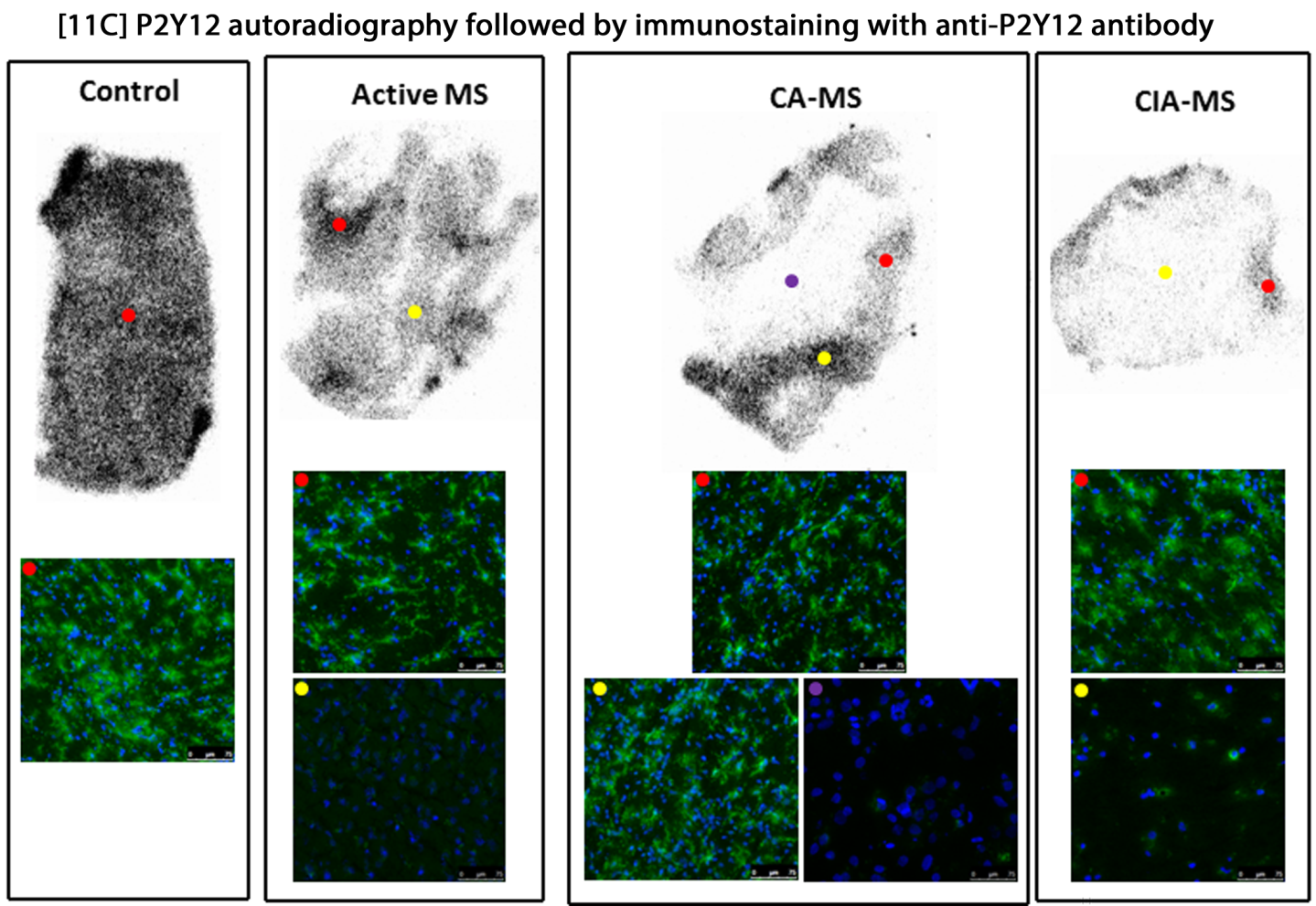


**Figure S18.** **Post [^11^C]P2Y12R-ant autoradiography staining with P2Y12R antibody on human MS tissue.** After autoradiography experiment with [^11^C]P2Y12R-ant tracer, sections were fixed with acetone and immunostaining was performed with P2Y12R antibody using the same staining protocol described in materials and methods. The high binding of the [^11^C]P2Y12R tracer colocalize with the high staining of the P2Y12R antibody (green) observed by immunofluorescence. Blue is nuclear staining with DAPI.


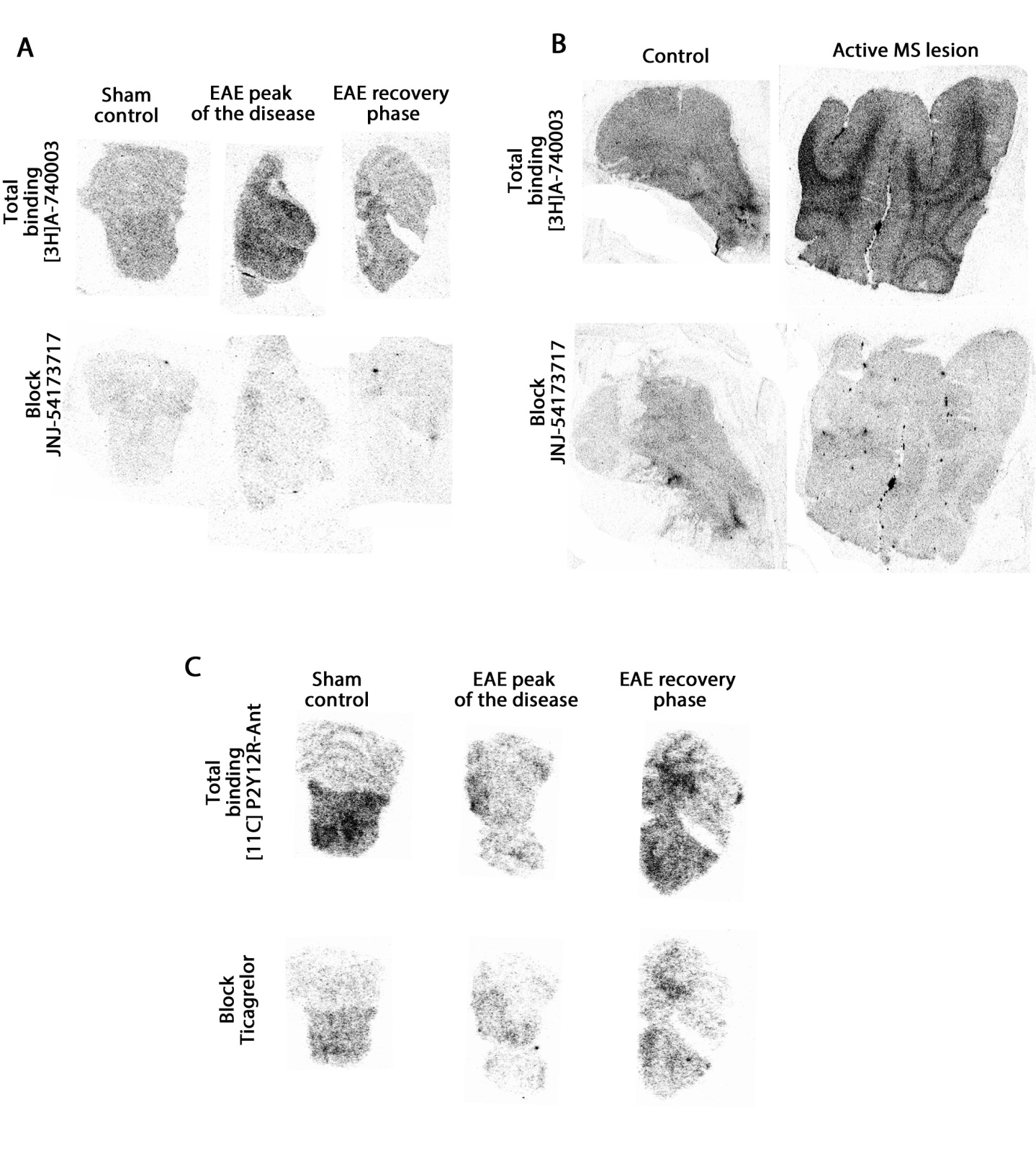


**Figure S19.** **Autoradiography blocking of P2Y12R ([^11^C] P2Y12-ant) and P2X7R radiotracers ([^3^H]A-740003) on acute rat EAE tissue and human MS tissue.** Blocking of [^3^H]A-740003 binding with JNJ-54173717 (10 µM) on rat EAE tissue **(A)** and human MS tissue **(B)**. Blocking of [^11^C] P2Y12-ant with Ticagrelor (10 µM) on rat EAE tissues **(C)**.

**Table S1. Multiple sclerosis patient details**

| **Case** | **Lesion characterization** | **Gender** | **Age** | **PMD (hrs:min)** | **Cause of death** |
| --- | --- | --- | --- | --- | --- |
| Patient 1 | Control | Male | 59 | 8:00 | Metastatic adrenal carcinoma |
| Patient 2 | Control | Female | 72 | 7:20 | Cardiac arrest |
| Patient 3 | Control | Female | 68 | 12:45 | Pulmonary embolism and heart failure |
| Patient 4 | Active | Male | 77 | 4:15 | CVA |
| Patient 5 | Active | Male | 51 | 11:00 | pneumonia |
| Patient 6 | Active | Female | 66 | 6:00 | unknown |
| Patient 7 | Chronic active | Female | 66 | 6:00 | unknown |
| Patient 8 | Chronic active | Male | 56 | 10:10 | pneumonia |
| Patient 9 | Chronic inactive | male | 70 | 7:45 | Cardiac arrest |
| Patient 10 | Chronic inactive | Female | 48 | 11:40 | Hepatic encephalitis |
| Patient 11 | Chronic inactive | Female | 64 | 7:45 | unknown |

**Table S2. List of primers used in this study**

| **Target gene** | **Primer sequence** |
| --- | --- |
| P2Y12R | Forward primer : ACCAGAGACTACAAAATCACCC  Reverse primer: AGAAAATCCTCATCGCCAGG |
| P2X7R | Forward primer : GAACAATATCGACTTCCCCGG  Reverse primer: TTATCGCCTGTTTCTCGGAAG |
| TSPO | Forward primer : CATCTACTCTGTCGTGGGCTA  Reverse primer: CAGACCAGGGTTGTGGACT |
| CXCL10 | Forward primer : TTCAAGGAGTACCTCTCTCTAG  Reverse primer: CTGGATTCAGACATCTCTTCTC |
| CD40 | Forward primer : CAAATACTGCGACCCCAACCTA  Reverse primer: TTTCTGAGGTGCCCTTCTGCT |
| TNF-α | Forward primer : AAACAACCCTCAGACGCCACAT  Reverse primer: AGTGCTCATGGTGTCCTTTCCA |
| CCL22 | Forward primer : CGTGATTACGTCCGTTACCG  Reverse primer: AAGGTTAGCAACACCACGC |
| CD206 | Forward primer : GTCTTGGGCCACAGGTGAA  Reverse primer: AAGGCGTTTGGATAGCCACA |

**Table S3. List of antibodies used for immunohistochemistry and immunofluorescence**

| **Primary antibody** | **Catalogue number** | **Company** | **Dilution** | **Incubation time** |
| --- | --- | --- | --- | --- |
| Polyclonal rabbit anti-human P2Y12R (recognize an epitope on the C-terminal) | 55042A | Anaspec | 1/50 | Overnight at 4 C |
| Polyclonal rabbit anti-mouse P2Y12R (recognize an epitope on the C-terminal) | 55043A | Anaspec | 1/500 | Overnight at 4 C |
| Monoclonal mouse anti-human P2X7R (recognize amino acids 81-106 within an internal region of human P2X7) | sc-514962 | Santa Cruz | 1/50 | Overnight at 4 C |
| Polyclonal rabbit anti-human P2X7R (recognize an epitope on the extracellular loop) | APR-008 | Alomone | 1/200 | Overnight at 4 C |
| Monoclonal mouse anti-rat ED1 | Clone ED1 | Gift | 1/200 | 60 min at RT |
| Monoclonal mouse anti-rat CD11b | Clone ED7 | Gift | 1/200 | 60 min at RT |
| GFAP-Cy3 | C9205 | Sigma | 1/300 | 60 min at RT |
| CD31 | M0823 | Dako | 1/50 | 60 min at RT |
| MHC-II | LN3 | Gift from pathology department at VUmc, Amsterdam | 1/500 | 60 min at RT |
| CD3 | M7254 | Dako | 1/50 | Overnight at 4 C |
| TSPO | LS-B5755 | Lifespan | 1/50 | Overnight at 4 C |
| **Secondary antibody** |  |  |  |  |
| Goat anti-rabbit IgG-Alexa488 | A11008 | Molecular probes | 1/400 | 60 min at RT |
| Goat anti-rabbit IgG-Alexa555 | A21429 | Molecular probes | 1/400 | 60 min at RT |
| Goat anti-mouse IgG-Alexa488 | A11029 | Molecular probes | 1/400 | 60 min at RT |
| Goat anti-mouse IgG-Alexa555 | A21424 | Molecular probes | 1/400 | 60 min at RT |
